# An integrated genetic linkage map for silkworms with three parental combinations and its application to the mapping of single genes and QTL

**DOI:** 10.1186/1471-2164-10-389

**Published:** 2009-08-21

**Authors:** Shuai Zhan, Jianhua Huang, Qiuhong Guo, Yunpo Zhao, Weihua Li, Xuexia Miao, Marian R Goldsmith, Muwang Li, Yongping Huang

**Affiliations:** 1 Institute of Plant Physiology and Ecology, Shanghai Institutes for Biological Sciences, Chinese Academy of Sciences, Shanghai, 200032, PR China; 2 Sericultural Research Institute, Chinese Academy of Agriculture Sciences, Zhenjiang, 212018, PR China; 3 Biological Sciences Department, University of Rhode Island, Kingston, RI 02881, USA

## Abstract

**Background:**

*Bombyx mori*, the domesticated silkworm, is a well-studied model insect with great economic and scientific significance. Although more than 400 mutations have been described in silkworms, most have not been identified, especially those affecting economically-important traits. Simple sequence repeats (SSRs) are effective and economical tools for mapping traits and genetic improvement. The current SSR linkage map is of low density and contains few polymorphisms. The purpose of this work was to develop a dense and informative linkage map that would assist in the preliminary mapping and dissection of quantitative trait loci (QTL) in a variety of silkworm strains.

**Results:**

Through an analysis of > 50,000 genotypes across new mapping populations, we constructed two new linkage maps covering 27 assigned chromosomes and merged the data with previously reported data sets. The integrated consensus map contains 692 unique SSR sites, improving the density from 6.3 cM in the previous map to 4.8 cM. We also developed 497 confirmed neighboring markers for corresponding low-polymorphism sites, with 244 having polymorphisms. Large-scale statistics on the SSR type were suggestive of highly efficient markers, based upon which we searched 16,462 available genomic scaffolds for SSR loci. With the newly constructed map, we mapped single-gene traits, the QTL of filaments, and a number of ribosomal protein genes.

**Conclusion:**

The integrated map produced in this study is a highly efficient genetic tool for the high-throughput mapping of single genes and QTL. Compared to previous maps, the current map offers a greater number of markers and polymorphisms; thus, it may be used as a resource for marker-assisted breeding.

## Background

Silk fibers are derived from the cocoon of the silkworm *Bombyx mori*, which was domesticated over the past 5,000 years from the wild progenitor *Bombyx mandarina*. Cocoon quality is very important because it can influence the yield of sericulture and determines whether a silkworm line can be used in silk production. Through the efforts of silkworm breeders over several thousands of years, many silkworm strains have been collected and conserved. Moreover, the different properties of these conserved silkworm strains, such as filament length, cocoon weight, cocoon shell weight, cocoon shell ratio, and cocoon color, have distinctive applications. Until now, crossbreeding was the only method of enriching loci that control cocoon quality to enhance the yield from a silkworm cocoon. Modern techniques involving gene cloning and marker-assisted breeding are now widely considered to be the most effective way of improving silk properties.

Genetic linkage map is an essential tool for mapping traits of interest and are used in positional cloning and marker-assisted breeding. Some genetic maps for the silkworm have been reported, including various genetic markers such as restriction fragment length polymorphisms (RFLPs; [1,2]), random amplified polymorphic DNA (RAPD; [3,4]), amplified fragment length polymorphisms (AFLPs; [5]), simple sequence repeats (SSRs; [6,7]), and single nucleotide polymorphisms (SNPs; [8,9]). SSRs (also called microsatellites) are generally accepted to be ideal markers because of their sound transferability, high reproducibility, and co-dominant inheritance. SSR markers are especially suitable for high-throughput genotyping, allowing rapid analysis of hereditary monogenetic traits and quantitative trait loci (QTL). Once SSR markers were established, polymorphisms could be detected merely by visualizing PCR products on an agarose gel, and these markers are still important for the meiotic analysis of livestock and agricultural species [10-15].

In our previous SSR linkage map [7], the 518 robust markers reported accounted for only 20% of all identified SSRs. The number of polymorphisms was low due to reliance on parental combinations between Dazao and C108 and to the minimal number of polymorphisms that occur between silkworm strains. Because the genetic distance between markers can be as large as 6.3 cM, fine mapping and gene cloning remain difficult.

A general approach for increasing the marker density in genetic linkage maps involves the identification of more markers and the integration of several linkage maps. Xia et al. [16] constructed an integrated, high-density linkage map of soybean using RFLPs, SSRs, sequence-tagged sites (STSs), and AFLP markers. Similarly, Vezzulli et al. [17] constructed an integrated map of grapevine using SSR and SNP markers. In many cases, the maps from different parental populations and even species have been integrated [18-20].

The choice of using the parental population of Dazao and C108 was based mainly on its internationally consistent use in silkworm genetic research. However, neither strain is applied widely for economic production of silk-related products. In China, more than 70% of silkworm breeders use the Jingsong strain for practical applications. Jingsong has properties that are advantageous for silk production, such as an average filament length of 1,200–1,500 m. In contrast, L10, which has poor silk-producing properties, possesses high stress resistance. Additional matings between strains of different origins may increase the mapping efficiency of markers due to the increased potential for genetic diversity.

**Figure 1:**
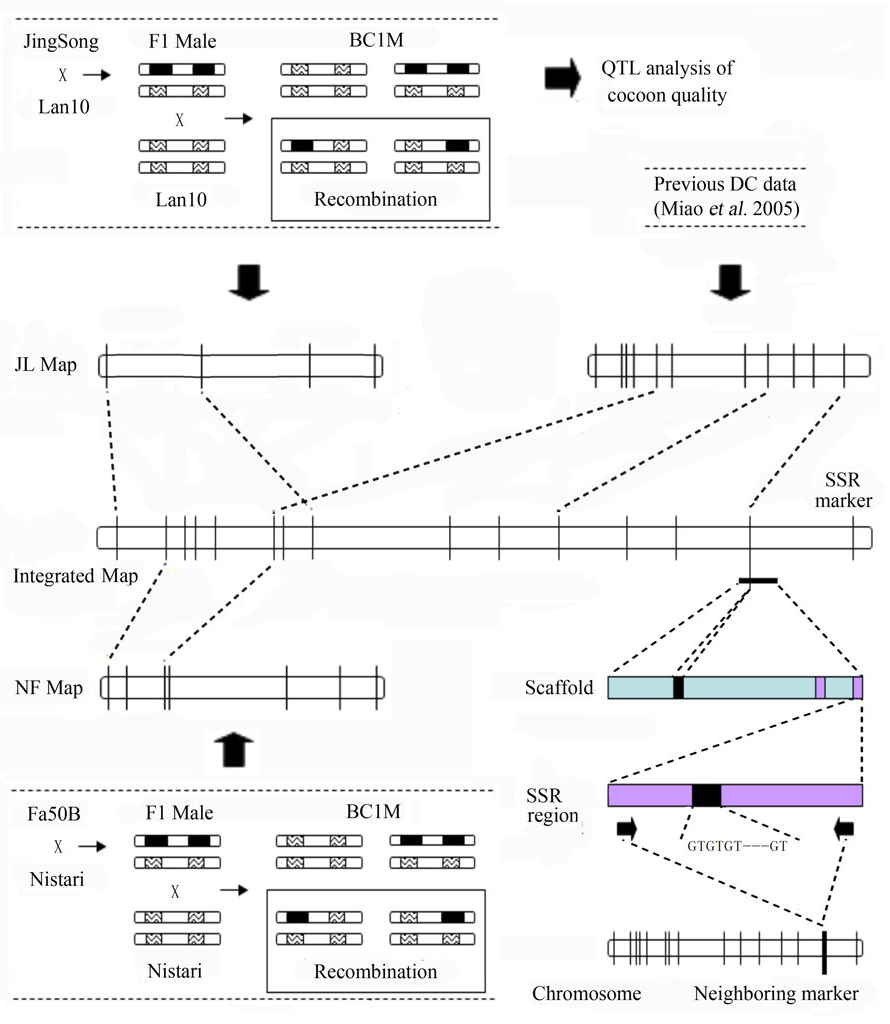
**Outline of the study**. We mapped SSR loci based on the mapping population Fa50B × Nistari and Jingsong × Lan10. Two new SSR linkage maps (NF and JL) were constructed, and the resultant data set was merged with previous data sets to generate the integrated linkage map. The mapped SSR sites were used to localize silkworm genomic scaffolds and SSRs were sought in the extended genomic sequences to develop neighboring markers. Thus, informative bins were formed by integrating the substitutes with the mapped sites.

Herein, we report an improved method for constructing silkworm SSR genetic maps with more informative loci based on new mapping populations (Figure 1). Using this approach, we localized QTL for whole cocoon weight, cocoon shell weight, cocoon shell ratio, and pupal weight. This work underpins the further cloning of genes that control properties advantageous to silk production and will be utilized further to identify molecular markers to assist in the breeding of productive silkworm lines.

## Results

### Markers in the linkage map

Approximately 2,670 SSRs isolated from our genomic libraries were subjected to polymorphism detection, including 518 SSRs that had been mapped [7]. In total, 271 markers were found to be polymorphic between Nistari and Fa50B, while 119 were polymorphic between Lan10 and Jingsong.

### Map properties

All polymorphic markers were genotyped in 188 segregants of the backcrosses between Fa50B × Nistari (NF, see Methods), or 190 of Lan10 × Jingsong (JL, see Methods). We analyzed the genotyping data from the inheritance pattern of BC_1_M populations. As a result, two new SSR linkage maps were constructed.

The linkage map constructed from the NF data consisted of 251 SSR markers (Table 1), which covered all silkworm chromosomes except 1 and 7. All of the maps for 27 LGs integrated with another fourteen gene-based loci are shown in additional file 1, 2, 3 and 4. The total map length spanned 1,859 cM with an average density of about one marker per 7 cM.

**Table 1:** Summary of each chromosome in the integrated map

		Mapped Sites					Neighboring Markers	
				
LG	Map Length (cM)		Source^1^					
					
		Int.	DC	NF	JL	Anch.^2^	Conf.^3^	Polym.^4^
1	51.4	8	6	0	4	2	9	4
2	123.6	22	14	11	0	3	18	13
3	178.9	26	18	9	2	3	22	15
4	86.8	22	21	3	4	5	20	10
5	106.5	23	18	13	0	8	14	4
6	92.5	15	11	7	4	5	7	2
7	78.9	12	12	0	0	0	9	4
8	145	26	20	10	6	6	15	11
9	76.8	19	13	8	0	2	16	9
10	129.2	25	22	5	0	2	28	18
11	140.4/32.7	45/8	38	19	7	11	30	17
12	167	24	20	8	2	5	20	9
13	120.6	26	14	11	8	7	10	3
14	108.4	32	25	13	2	8	22	15
15	79.2	19	12	11	0	4	16	7
16	72.9	22	13	10	5	4	8	1
17	99.8	17	9	8	3	2	12	2
18	137.5	28	20	13	4	8	20	10
19	73.2	20	14	7	3	4	17	7
20	131.3	26	20	6	5	5	15	8
21	203	19	17	4	2	4	15	9
22	117.3	25	17	10	3	4	19	10
23	109.5	35	22	16	6	7	32	14
24	167.4	46	32	17	2	5	32	12
25	121.3/41.8	29/6	23	10	6	5	16	9
26	179.9	30	25	9	2	5	19	9
27	133.1	21	18	2	6	3	8	2
28	89	18	16	4	3	5	16	4
X	+40.9	-	-	5	-	-	3	2

Total^5^	3320.7/3068.1	692	510	244	89	132	485	238

1 Paradoxical markers were removed when integrating; only the consensus data were reserved.

2 Anch.: anchor marker, genotyped in more than one cross

3 Conf.: confirmed neighboring marker

4 Polym.: polymorphic neighboring markers

5 The total does not include information from Chr. X.

Whereas the linkage map constructed from the JL data consisted of 94 markers (Table 1), they were linked to 24 groups and assigned to 23 chromosomes in the JL map. The number of markers on the linkage groups ranged from two to eight (see additional file 5 and 6). The genetic distance ranged from 0.8 to 158 cM in length, and the total length of the SSR linkage map was 1,181.5 cM.

Information related to the markers mentioned above is provided in additional file 7.

### Map integration

We collected Dazao × C108 (DC, see Methods) genotyping data for map integration. Markers in the merged data set were divided into 30 LGs and directly linked to 28 chromosomes according to the known sites. Both Chr. 11 and Chr. 25 corresponded to two LGs, but they only shared one anchor between the pair of LGs. As determining the interposal direction corresponding to the framework sequence was difficult, we could not construct consensus maps for these two chromosomes. Nevertheless, we established a relationship for the two pairs of separated groups via single progeny mapping.

**Figure 2:**
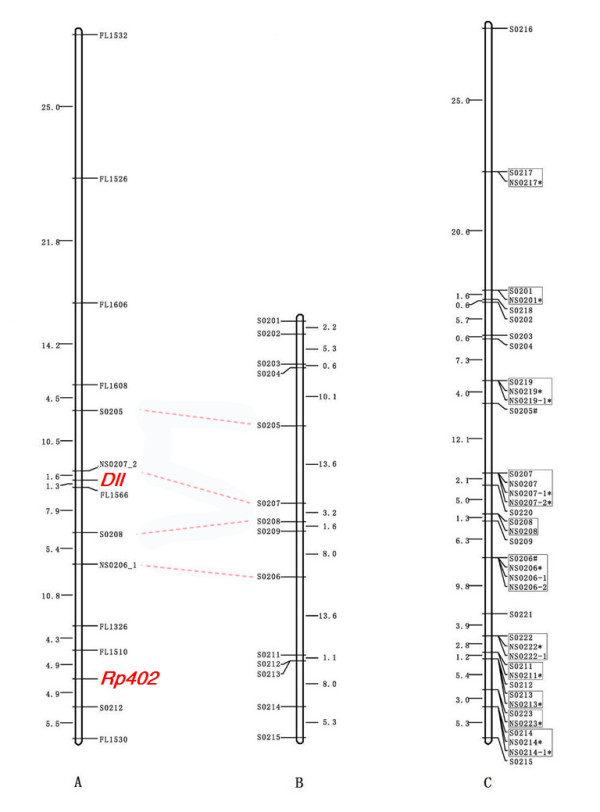
**Single progeny and integrated maps for Chr. 2**. (A) The most likely NF map was constructed for Chr. 2. The font name in red represents gene-based markers. The names of sites beginning with "FL," "S," and "NS" represent unreported markers, previously assigned markers, and neighboring markers, respectively. (B) The DC map for Chr. 2 was modified according to the likelihood generated by a previous data set [7]. As a result, the order from S0206 to S0209 was different from that in the previous report. In addition, four pairs of sites aligned by dotted lines reveal a consistency between the mapping results generated by original markers and their neighboring markers. (C) The final integrated linkage map for Chr. 2, in which the sites were named by a uniform format "S02XX," following the previous mapped sites in this chromosome. The neighboring markers (referred to as "NS02XX") are included in the box with their original sites. The NF, JL, and integrated maps for all of the chromosomes are listed in Figures S1, S2, and S3.

The completely integrated map contained 692 unique SSR sites, with a total map length of 3,320 cM (Figure 2; additional file 8, 9, 10, 11 and 12; Table 1). Three markers (S0604, S0613, and S2115) differed significantly between the crosses and were therefore excluded during integration. S2119 and S2601 were placed at the ends, far from the other loci (≥ 40 cM), because attached Cleaved Amplified Polymorphic Sequences (CAPS) or gene-based markers were not included in map integration; two end markers (S2103 and S1817), however, reserved in the NF populations were no longer obvious when integrated with the other data sets. This suggests that gaps present in a single map can be filled by integration with other data sets. The length of the integrated map was even shorter than that of the map of the DC populations, which contained 548 sites. The mean inter-locus distance of 4.8 cM shows that the integrated map has a higher density (the greatest density was ~3 cM per locus on Chr. 11).

### Application of the map to locus mapping

**Figure 3:**
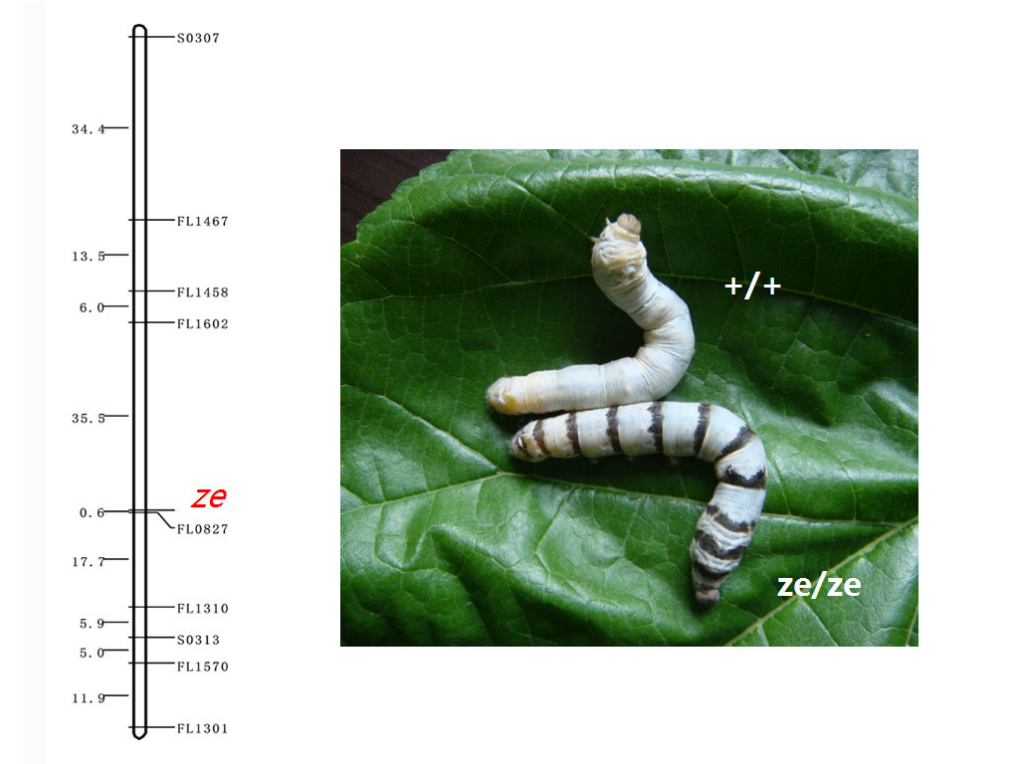
**Mapping results for the Ze locus**. The Ze locus, which controls the zebra-like stripes of larvae, was preliminarily mapped to Chr. 3. The picture on the right shows the phenotypes of wild-type and Ze-mutant strains.

The Zebra locus was mapped to Chr. 3, consistent with previous data [21]; moreover, we found a tightly linked marker (FL0667) with a map distance of 0.6 cM (Figure 3). We also located a number of silkworm genes in this new mapping population. *Dll *was positioned near FL1556 (Figure 2A), while twelve ribosomal protein genes (Rp genes) were located in nine linkage groups; the map distance to their nearest markers ranged from 0 to 14.5 cM (Table 2). Hence, SSR linkage maps appear to be a useful and reliable tool for locating functional genes.

**Table 2:** Detailed information for the thirteen mapped genes (the gene-based markers used for mapping are listed in additional file 13)

Locus name	Accession number	Gene description	LG	Position
Rp0201	AY769345	ribosomal protein S29	25	14.5 cM to S2508
Rp2001	AY769324	ribosomal protein S10	5	6.6 cM to Rp5401
Rp2501	AY769319	ribosomal protein S5	22	0 cM to S2206
Rp2602	AY769318	ribosomal protein S4	21	8.6 cM to S2120
Rp2701	AY769317	ribosomal protein S3A	17	3.7 cM to S1709
Rp2902	AY769315	ribosomal protein S2	13	1.1 cM to S1325
Rp3401	AY769310	ribosomal protein L38	17	3.3 cM to S1701
Rp402	AY769343	ribosomal protein S27A	2	4.9 cM to S0212
Rp5401	AY769289	ribosomal protein L19	5	2.1 cM to S0523
Rp5701	AY769286	ribosomal protein L17	21	7 cM to S2119
Rp6801	AY769275	ribosomal protein L7A	15	4.4 M to S1509
C41	AY769270	ribosomal protein L4	11	3.7 cM to S1120
Dll	-	*dll*	2	1.3 cM to S0220

**Figure 4:**
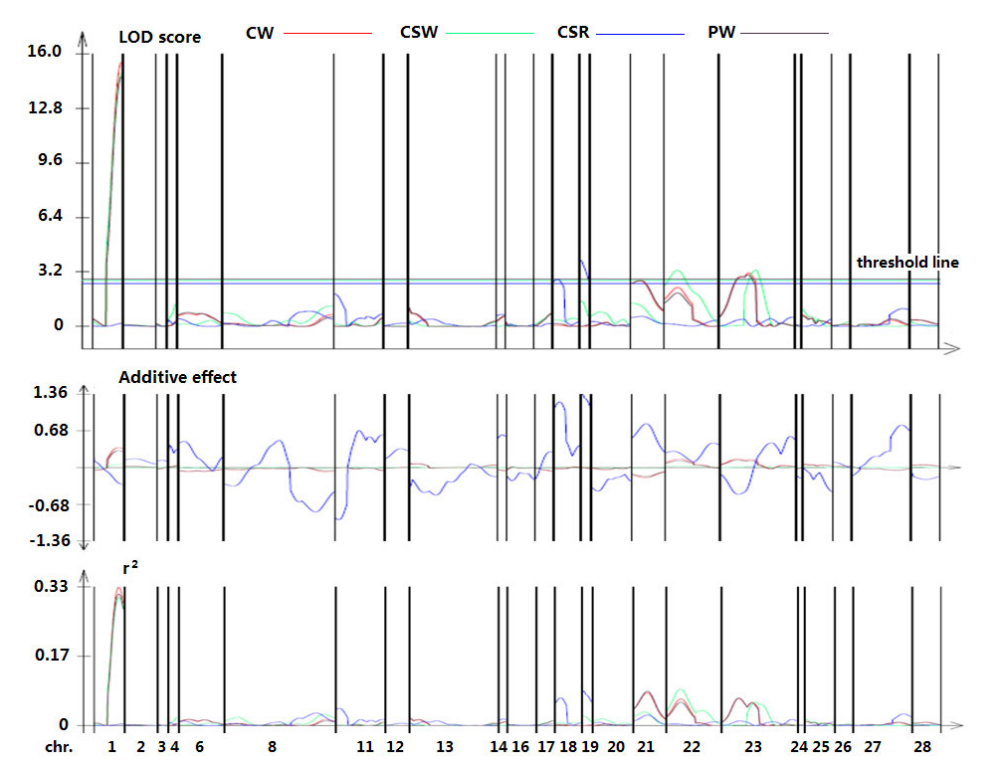
**Graphical overview of our QTL mapping results**. From top to bottom, the three graphs show the LOD scores, additive effects, and r^2 ^values for each trait (each in a different color). Each chromosome is indicated by vertical lines, with the chromosome numbers along the bottom. In the LOD score graph, a threshold line is indicated for each trait based on the threshold values. Peaks above the threshold line indicate QTL.

Based on the JL data set, we identified twelve candidate loci involved in whole cocoon weight (CW), cocoon shell weight (CSW), cocoon shell ratio (CSR), and pupal weight (PW) (Figure 4, additional file 13). Of these, six QTL were confirmed by independent analysis (Table 3). Q1 on Chr. 1 had the most significant contribution for the three traits, accounting for 29.38, 27.75, and 27.96% of the phenotypic variation in CW (LOD = 15.49), CSW (LOD = 14.85), and PW (LOD = 14.64). The other putative QTL (Q2 and Q3) associated with CW and PW had relatively weak effects (LOD of about 3), which were localized in the neighboring region of S2304. Q4 for CSW was mapped to Chr. 23, which also contributed a relatively small effect (r^2 ^= 5.23%, LOD = 3.30). For CSR, two QTL (Q5 and Q6) were identified on Chrs. 18 and 19 that accounted for 6.54 and 8.28% of the phenotypic variance, respectively.

**Table 3:** Putative major QTL and their genetic effects on whole cocoon weight and related traits from BC1M of JL (100 replicates)

Trait	QTL	Chr.^1^	Position (cM)	LOD^2^	Additive effect	r^2 ^(%)	Power ^3 ^(%)
Cocoon weight	Q1	1	41.81	15.49	0.35	29.38	100
		21	12.01	2.68	-0.18	6.68	7
	Q2	23	32.01	2.94	0.14	5.19	28
	Q3-1^4^	23	41.61	3.15	0.15	5.54	28

Cocoon shell weight	Q1	1	41.81	14.85	0.05	27.75	92
		22	18.01	3.31	0.03	8.71	6
	Q4	23	51.61	3.30	0.02	5.23	49

Cocoon shell ratio	Q5	18	6.01	2.77	1.21	6.54	96
	Q6	19	2.01	3.89	1.36	8.28	100

Pupal weight	Q1	1	41.81	14.64	0.30	27.96	100
	Q2	23	32.01	2.91	0.12	5.23	29
	Q3-2^4^	23	39.61	3.03	0.12	5.27	29

1 Chromosome

2 The thresholds of LOD for CW, CSW, CSR, and PW were 2.67, 2.69, 2.54, and 2.78, respectively.

3 The statistical power represents the detected number of LOD values that were > 2 in 100 random replicates by multi-marker joint analysis.

4 Q3-1 possesses an extremely similar LOD value (LOD = 3.02) for PW to Q3-2. In addition, their similar effects (r^2 ^of 13.95 and 13.94%) and close positions (2 cM) indicate that they may share the same region.

We also tested for epistatic genetic effects in our mapping population. A total of 20 pairs of loci had significant statistical power for CW, PW, and CSR (additional file 14). Most epistatic pairs involved CSR, which lacked major QTL relative to the other three traits. This suggests that CSR may possess more complex genetic properties than the other traits.

### Neighboring marker development

Due to the low frequency of polymorphisms, SSR markers are limited when mapped in other strains [6,7]. The emergence of genomic sequences for the silkworm [22,23] offered an opportunity to find substitutes in the extended region adjacent to mapped sites, and the resulting bins provided informative sites in the map. Specifically, 477 sites were linked to draft sequences for extension, most of which were typed in only one cross. We designed 857 pairs of primers flanking the SSR regions for 456 sites, and 708 markers were analyzed for amplification efficiency and polymorphisms in a test panel comprising six representative strains (Dazao, C108, Jingsong, Lan10, Fa50B, and Nistari). Of these, 497 markers yielded reliable and distinctive bands, and 244 exhibited more than one band pattern. All of the confirmed neighboring markers that integrated with their original site are shown in additional file 8, 9, 10, 11 and 12. Additional file 15 contains the primer sequences applied in this study.

To verify the reliability of the neighboring markers, we randomly selected two pairs of polymorphic markers: NS0206/NS0207 from DC and NS2329/NS2333 from NF, which were genotyped in NF and DC, respectively. As illustrated in Figure 2B, the orders generated by substitution were consistent with our original results.

## Discussion

### Mapping efficiency of the population

The backcross of Dazao and C108 has been used to produce the major mapping population by several groups [1,4,8,24]. However, the density of genetic markers is generally low given the chromosome number and genome content of the silkworm. For consistency, our group has also selected this mapping population to construct the first SSR linkage map [7]. Although, we attempted to identify more markers, the low incidence of polymorphisms (22.2%) remains a major limitation that impedes the augmentation of marker density. Therefore, we selected two other parental populations, Jingsong × Lan10 and Fa50B × Nistari, for combination. The integration of the three combinations (DC, JL, and FN) allowed more than 150 new markers to be anchored in the integrated map.

Although the number of markers increased, it was below our expectations. The 518 markers that were initially mapped (20% of all that were identified) from DC had a high percentage of polymorphisms compared to other sources (40% of those mapped by NF and 75% by JL; additional file 16). To some extent, this explains the relatively low number of new markers identified in this study: once an SSR locus was identified in one mapping population, it was more likely to be mapped in the other populations. This transitivity of polymorphisms indicates that the polymorphic loci are shared by several silkworm strains. On the other hand, the two pairs of parents used in this study had large genetic differences. However, less than 20% of the SSR markers could be mapped. These results indicate that although *B. mori *differentiated from *B. mandarina *more than 5,000 years ago, their genomes are still highly homologous. Additionally, we believe that Dazao × C108 is the most useful population for genotyping genetic markers.

### An effective strategy for developing polymorphic markers

Because the efficiency of markers is low for silkworm genotyping, we developed new markers based on genomic sequences and tried to include more available information for mapping in different populations. We considered SSRs to be relatively good candidates, given the increased frequency of polymorphisms compared with other sequence-based markers (Additional file 16). We therefore investigated the distribution of SSRs in 13.8 Mb of the silkworm genome for use in the development of neighboring markers. We predicted that certain types of SSRs would have a higher frequency of polymorphisms; thus, we sought to develop an effective strategy for identifying polymorphic markers. In this regard, the repeat motif and repeat number were taken into account.

**Figure 5:**
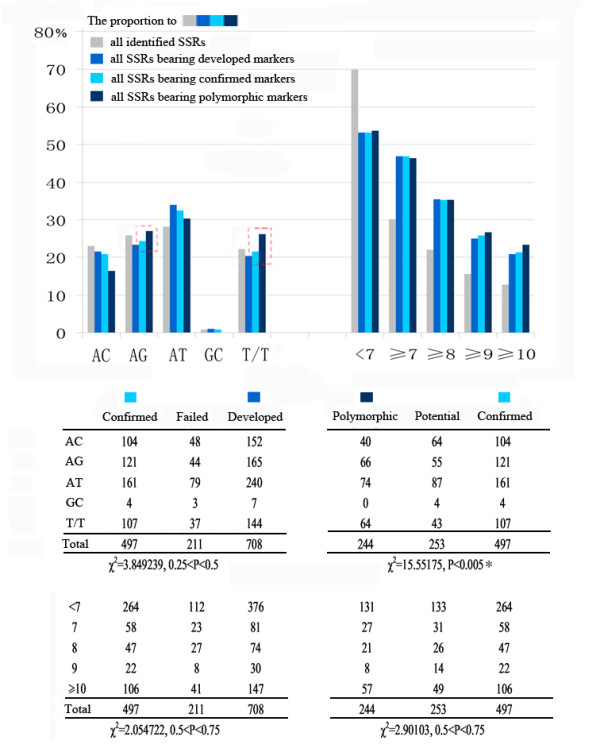
**Analysis of the relationship between SSR type and marker efficiency**. The enrichment analysis result for each type is shown in the bottom table while the colored bars in the top graph indicate the percentage of each type in the corresponding condition: blue bars indicate the frequency of each type of SSR based on all of the candidate SSRs used for marker development; light blue bars indicate the frequency of each SSR type based on all of the confirmed markers (i.e., those that amplified the product successfully); dark blue bars indicate the frequency of each SSR type to all of the polymorphic markers. A correlative analysis was performed for the four data sets. Our χ^2 ^test results are shown at the bottom of the table; significant correlations are indicated by a star.

A total of 2,903 SSRs were identified and categorized into five groups based on their motif type: (AC)n, (AG)n, (AT)n, (GC)n, and T/T ("T/T" for tri- and tetra-nucleotides). The distribution of SSRs among the five types was 23, 25.8, 28.1, 0.9, and 22.2%, respectively (see the gray bars in Figure 5). The abundance of (GC)n was low significantly in accordance with that reported in silkworm [6] and other organisms [25-28]. Next, we performed the enrichment analysis for each type of SSR in the confirmed markers and polymorphic markers, respectively. An analysis by χ^2 ^testing indicated that the type of motif had a significant effect on the frequency of polymorphisms (*P *< 0.005), but not on product amplification (Figure 5). It is accepted that amplification ability is related to the flanking sequences (bearing the primers) rather than to the core repeat units, whereas the structure of the motif might affect sequence stability following replication. The (AG)n and T/T motifs were more polymorphic than the other motif types (see the red boxes in Figure 5). For repeat number, we utilized a similar statistical method. A large number of highly repetitive SSRs were identified as candidate markers, which revealed our priority for SSRs with a longer repeat region. However, no correlations were found between the repeat number and amplification efficiency or frequency of polymorphisms (Figure 5).

While lacking a detailed explanation in terms of mechanism, our analysis suggests that the frequency of polymorphisms among the SSRs was related to their motif type, and that SSRs with an (AG)n or T/T motif are good candidates for the development of polymorphic markers.

### Distribution of SSRs in the silkworm genome

We next conducted an investigation into all available silkworm scaffolds [29], corresponding to 344 Mb, in order to determine the distribution of the SSRs. A total of 9,426 scaffolds were used to identify 21,122 objects with a repeat number > 6 (Table 4). Of these, the number of objects detected in 4,226 of the scaffolds ranged from 1 to 18.

**Table 4:** Summary of SSRs distributed in all silkworm genomic scaffolds

No. of repeats	≥ 6	≥ 7	≥ 8	≥ 9	≥ 10
Scaffold	9,426	6,809	5,046	3,763	2,893
SSR	21,122	11,788	7,679	5,238	3,782
AC (%)	4,024 (19.1%)	2,486 (21.1%)	1,729 (22.5%)	1,243 (23.7%)	933 (24.7%)
AG (%)	4,707 (22.3%)	3,268 (27.7%)	2,426 (31.6%)	1,800 (34.4%)	1,349 (35.7%)
AT (%)	5,969 (28.3%)	3,237 (27.5%)	2,182 (28.4%)	1,521 (29.0%)	1,120 (29.6%)
CG (%)	56 (0.27%)	8 (0.07%)	4 (0.05%)	3 (0.06%)	3 (0.08%)
T/T^1 ^(%)	6,366 (30.1%)	2,789 (23.7%)	1,338 (17.4%)	671 (12.8%)	377 (10.0%)
Details of T/T^1^					

AT-rich (%)	5,979 (93.9%)	2,627 (94.2%)	1,230 (91.9%)	590 (87.9%)	313 (83.0%)
Most tri-	AAT (2,589)	AAT (1,047)	AAT (441)	AAT (181)	ATC (79)
	ACT (976)	ACT (516)	ACT (269)	ATC (133)	AAT (73)
	ATC (923)	ATC (499)	ATC (262)	ACT (130)	ACT (67)
Most tetra-	AAAT (545)	AAAT (216)	AAAT (110)	AAAT (70)	AAAT (43)
	ACAT (74)	AAAG (43)	AAAG (35)	AAAG (24)	AAAG (19)
	AAAG (65)	ACGC (37)	ACGC (28)	ACGC (23)	ACGC (18)

1 T/T represents the repeat units that comprise tri- and tetra-nucleotides.

A number of interesting observations were made concerning the genome. For example, (AT)n constituted ~40% of the di-nucleotides (28.3% of the total), while (A+T)-rich repeats had an absolute majority among the tri- and tetra-nucleotide types with a large number of repeats (Table 4). This dominance has not been observed in other organisms [25,30]. Further investigation of the silkworm genome revealed that among those motifs with > 3 bases, it is somehow difficult to form long SSRs (Table 4). In accordance with the dominance of (A+T)-rich sequences, the percentage of (AT)n also decreased as the repeat number increased; in turn, (AG)n was found to be relatively capable of forming long SSRs.

### Analysis of cocoon quality using QTL

Cocoon quality is an important characteristic in silkworms; however it is more difficult to map than single-factor Mendelian traits. In our study, we identified at least six QTL involved in the following traits: CW, CSW, CSR, and PW. Of these, Q1 had simultaneous effects on CW, CSW, and PW with a significant LOD score and phenotypic variance. Thus, this locus may be valuable for filament research. The other putative QTL (Q2–Q4) for CW, CSW, and PW were distributed in the interval between S2304 and FL1203. Q2 and Q3 actually represent the double peak of the LOD score from the QTL search for CW and PW, while Q4 represents another peak of CSW at a neighboring position (Figure 4). Although these QTL were just above the threshold values, multi-marker joint analysis revealed a relationship between these traits and S2304 or FL1203 (additional file 13), confirming that this continuous region has an effect on CW, CSW, and PW.

On the other hand, the genetic correlations between CW and CSW, CW and PW, and CSW and PW were extremely high (0.89, 0.99, and 0.85, respectively). The clustered distribution of the QTL is suggestive of the genetic relatedness of these traits and is consistent with data showing relationships between cocoon, cocoon shell, and pupal weight.

## Conclusion

The integrated linkage map described here has a greater number of sites and more than one optional marker for most sites, and is more efficient for a range of applications. In combination with recent silkworm genomic data [31], the fine mapping and positional cloning of interesting traits will be realized more easily. The identification of target genes will in turn facilitate detailed research of insect innate immunity, metamorphosis, hormone metabolism, and the genetic improvement of economical strains with high stress resistance.

## Methods

### Silkworm strains and mapping reagents

Two populations (Figure 1) were generated from four silkworm strains: Nistari (Indian origin), Fa50B (French origin), Jingsong (Chinese origin), and Lan10 (Chinese origin). Nistari and Fa50B have quite different origins, while Jingsong and Lan10 differ in terms of their ecological features [32]. These strains were preserved in the Sericultural Research Institute at the Chinese Academy of Agricultural Sciences. The 188 segregants of a single-pair backcross (BC_1_M) between a Nistari female and an F_1_male (Nistari female × Fa50B male) were used to genotype markers while another 22 segregants of a single-pair backcross (BC_1_F) between a Nistari male and an F_1 _female (Nistari female × Fa50B male) were used to validate the results of grouping, owing to a lack of crossing over in females [33]. A phenotypic trait of the Zebra locus (*Ze*^+^/*Ze*) was involved in the arrangement of the mapping panel, which causes narrow black bands on the anterior portion of each larval segment and dark brown cuticles on both sides of the head. The panel, including 94 BC_1_M individuals, without this trait was the Ze^+ ^panel, and the panel with this trait was the Ze panel. The other population was generated from (Jingsong × Lan10) F_1 _males backcrossed with Lan10 females. The materials used in this SSR investigation were randomly selected from 190 individuals in a single BC_1_M population. Each cocoon, cocoon shell, and pupa was weighed, and the cocoon shell ratio was calculated as CSW/CW.

Silkworm genomic DNA was extracted from individual fifth instar larvae (day 3) using previously described methods [4] and stored in 96-well PCR plates at a concentration of 20 ng/μL.

### Marker design

Markers for map construction were isolated from our genomic libraries [7]. Silkworm genomic scaffolds and ribosomal protein gene sequences were obtained from GenBank [29]. Gene-based markers were designed based on intron sequences.

Neighboring SSR markers were designed on flanking sequences bearing > 12 bp of core simple repeats, which involved 2–4-bp unit tracts. Python scripts were used for hunting SSRs from genomic sequences. SSRs with large numbers of repeats were preferred.

The primers were designed manually using the following criteria: 56–63°C for annealing, 40–60% GC content, and 100–500-bp products.

### Polymorphism survey and genotyping

SSRs were amplified by standard PCR following a Touchdown procedure [7]. For polymorphism detection, products were separated by polyacrylamide gel electrophoresis (8% non-denaturing gel in 1× TBE buffer at 110 V for 8 h). The detection of neighboring markers was carried out on an agarose gel (3% gel in 1× TAE buffer at 80 V for 60–80 min), considering the potentially broad application.

Markers for genotyping were 5'-labeled with FAM, HEX, TAMRA, or TET on the forward primer, and high-throughput typing results for segregants in BC_1_M and BC1F were obtained on an ABI-Prism 377 automated sequencer (5% denaturing polyacrylamide gel in 1× TBE buffer at 3000 V for 2 h; Applied Biosystems, Foster City, CA). Genotype patterns were visualized using Genescan^® ^3.1.2 (Applied Biosystems). The corresponding scoring was interpreted in two independent readings.

### Linkage analysis and map construction

The primary analysis, including a chi-square test and grouping, was carried out by JoinMap 3.0 [34]. According to the Mendelian ratio of 1:1 (3:1 for Z chromosome), loci with significant differences (*P *≤ 0.05) were discarded. The remaining loci were dissected into groups at increasing stringency levels of the linkage test (LOD values from 3 to 12). The final grouping results refer to the genotypes in BC1F.

MAPMAKER/EXP 3.0b [35] was then used to order valid loci for each group. We directly performed exhaustive comparative analysis to obtain the most likely order for groups with less than eight loci. To determine the arrangement of a larger group, we ordered the loci at least three times and accepted the consistent subset order. Where necessary, we attempted to find the most likely intervals for the remaining unplaced loci or to compare all possible orders for indefinite regions. In addition, we mapped the complete order with a calculated Kosambi distance for each group.

WinQTLCart 2.5 [36] was used to locate QTL in the Jingsong × Lan10 BC_1_M population for whole cocoon weight, shell weight, cocoon shell ratio, and pupal weight by composite interval mapping. For each trait, 1,000 permutations were performed to determine the threshold value, then the entire chromosome was scanned every 2 cM for the presence of QTL using a standard model. The positions of the candidate QTL were fixed based on the peak LOD scores.

To confirm the results generated by WinQTLCart 2.5 and to identify epistatic QTL, we carried out multi-marker joint analysis as shown below. The phenotypic value of the *i*th BC_1_M sample, *y*_*i*_, may be described by the following model:



where *μ *is the mean total; *m *is the number of markers; *a*_*k *_is additive effect for the *k*th marker (or QTL); *a*_*rs *_is the epistatic effect between the *r*th and *s*th markers; *x *represents dummy variables; and *ε*_*i *_is the residual error with an assumed *N*(0, *σ*^2^) distribution. One hundred imputed data sets for the marker genotypes were sampled at random using the conditional probabilities of the marker genotypes. Each data set was then analyzed by the penalized maximum-likelihood method [37]. Significant candidate loci were defined as having a statistical power above 20%.

### Mapping data set integration and consensus map construction

Two data sets generated by NF [Nistari × (Nistari × Fa50B)] and JL [Lan10 × (Jingsong × L10)] were integrated with the previous DC data set [C108 × (Dazao × C108), which included 189 individuals] [7]. We corrected some obvious errors and re-analyzed the previous data setset using a uniform rule. Locus order as determined by DC and NF crossing was considered the key reference for the integrated map. We included the JL data without regard to the linkage results.

Since MAPMAKER only handles one cross at a time, we could not directly pool scoring in different crosses to construct a consensus map. Instead, we first merged the single data sets into a consensus data set. Given the similar sizes of three mapping populations (189 individuals in DC, 190 in JL, and 188 in NF), we made up a large population of 567 individuals that could include three sets of scoring. From this, the first 189, the next 190, and the last 188 represented individuals came from DC, JL, and FN, respectively. If the individuals were not genotyped in the corresponding cross, they were considered as missing data loci (designated 0). Fortunately, we could treat many common markers, typed in two or three crosses with little or no missing data loci, as anchors for groups from different mapping populations. The resulting consensus data set was used to perform a linkage analysis using MAPMAKER. The framework generated by a single cross was accounted for if the result of ordering was dramatic. JoinMap was also employed for reference, which automatically constructs an integrated consensus map.

## Abbreviations

SSR: simple sequence repeats; QTL: quantitative trait loci; CAPS: cleaved amplified polymorphic sequences; CW: whole cocoon weight; CSW: cocoon shell weight; CSR: cocoon shell ratio; PW: pupal weight.

## Authors' contributions

YH, ML, XM, and SZ planned the work. ML arranged the strain mating. QG and WL prepared the DNA samples. SZ and XM designed the markers. SZ, QG, YZ, and WL carried out the genotyping. SZ, JH, and ML performed the data analysis. SZ and ML wrote the draft. YH, MRG, and JH commented on an earlier draft. All authors have read and approved the final draft of the manuscript.

## Supplementary Material

Additional file 1 **NF linkage maps, part I**. The most likely linkage maps for seven chromosomes (2, 3, 4, 5, 6, 8 and 9) generated by the NF data set are shown. Click here for file

Additional file 2 **NF linkage maps, part II**. The most likely linkage maps for seven chromosomes (10, 11, 12, 13, 14, 15 and 16) generated by the NF data set are shown. Click here for file

Additional file 3 **NF linkage maps, part III**. The most likely linkage maps for seven chromosomes (17, 18, 19, 20, 21, 22 and 23) generated by the NF data set are shown. Click here for file

Additional file 4 **NF linkage maps, part IV**. The most likely linkage maps for six chromosomes (24, 25, 26, 27, 28 and the unassigned group X) generated by the NF data set are shown. Click here for file

Additional file 5 **JL linkage maps, part I**. The most likely linkage maps for ten chromosomes (1, 2, 3, 4, 6, 8, 11, 12, 13 and 14) generated by the JL data set are shown. Click here for file

Additional file 6 **JL linkage maps, part II**. The most likely linkage maps for 13 chromosomes (16, 17, 18, 19, 20, 21, 22, 23, 24, 25, 26, 27 and 28) generated by the JL data set are shown. Click here for file

Additional file 7 **Information on the markers used in this study**. This table contains the work name, map name, forward primer sequence, reverse primer sequence, labeled type, and population source. Click here for file

Additional file 8 **Integrated maps of three mapping populations, part I**. The most likely linkage maps for the chromosomes integrated with three data sets are shown in this file and the following four additional files (additional file 9, 10, 11 and 12). We removed five loci that showed significant disagreement in the distance to adjacent loci (S1401), position in the group (S1116 and S2711), or grouping result (S0210 and S1140). Furthermore, a number of markers with improved likelihood over the initial map appeared in seven groups: Chr. 2 (S0206–S0209; Figure 2B), Chr. 3 (S0312–S0315), Chr. 4 (S0403–S0404 and S0418–S0419), Chr. 9 (S0901–S0907), Chr. 18 (S1813–S1815), Chr. 20 (S2018–S2019), and Chr. 26 (S2617–S2622). Inverted orders were observed in Chr. 22 (S2209–S2210 and S2211–S2212) and Chr. 10 (S1012–S1013) because the CAPS marker was not included. The sites that contained confirmed neighboring markers are indicated by a star. An additional star indicates that a polymorphism was detected. Chromosomes 11' and 25' shared one common marker; however, their order could not be determined. This file contains the maps of six chromosomes (1, 2, 3, 4, 5 and 6). Click here for file

Additional file 9 **Integrated maps of three mapping populations, part II**. The most likely linkage maps for five chromosomes (8, 9, 10, 11 and 12) integrated with three data sets are shown. The sites that contained confirmed neighboring markers are indicated by a star. An additional star indicates that a polymorphism was detected. Chromosome 11 and 11' shared one common marker; however, their order could not be determined. The map of chromosome 7 is not included here because there were not any new identified markers on it. Click here for file

Additional file 10 **Integrated maps of three mapping populations, part III**. The most likely linkage maps for six chromosomes (13, 14, 15, 16, 17 and 18) integrated with three data sets are shown. The sites that contained confirmed neighboring markers are indicated by a star. An additional star indicates that a polymorphism was detected. Click here for file

Additional file 11 **Integrated maps of three mapping populations, part IV**. The most likely linkage maps for five chromosomes (19, 20, 21, 22 and 23) integrated with three data sets are shown. The sites that contained confirmed neighboring markers are indicated by a star. An additional star indicates that a polymorphism was detected. Click here for file

Additional file 12 **Integrated maps of three mapping populations, part V**. The most likely linkage maps for six chromosomes (24, 25, 26, 27, 28 and the unassigned group X) integrated with three data sets are shown. The sites that contained confirmed neighboring markers are indicated by a star. An additional star indicates that a polymorphism was detected. Chromosome 25 and 25' shared one common marker; however, their order could not be determined. Click here for file

Additional file 13 **Result of 100 replicates of multi-marker joint analysis in sample JL**. We included the major and epistatic QTL that were detected at least once in 100 replicates by multi-marker joint analysis (LOD = 2). The position information, power value, mean LOD score, mean effect value, and individual value of each replicate for the filtered loci are listed in the file. Click here for file

Additional file 14 **Putative epistatic QTL and their interaction effects (100 replicates)**. We performed multi-marker joint analysis to estimate the association between markers and phenotypes. This table contains the significant interacting pairs with their statistical power value (at least 20%), mean LOD score, and mean interaction effect in 100 replicate tests. Click here for file

Additional file 15 **Information on all confirmed neighboring SSR markers**. The marker name, original site, forward primer sequence, reverse primer sequence, and detection results are shown. Click here for file

Additional file 16 **Comparison of the efficiency of different mapping populations and marker types**. This table contains the origin of three populations and the counting information on markers used in each population. Click here for file
