# Supplementary figures and images for: An integrated genetic linkage map for silkworms with three parental combinations and its application to the mapping of single genes and QTL
Source: BMC Genomics. 2009 Aug 21;10:389. doi: 10.1186/1471-2164-10-389 (PMC2741490; doi:10.1186/1471-2164-10-389)

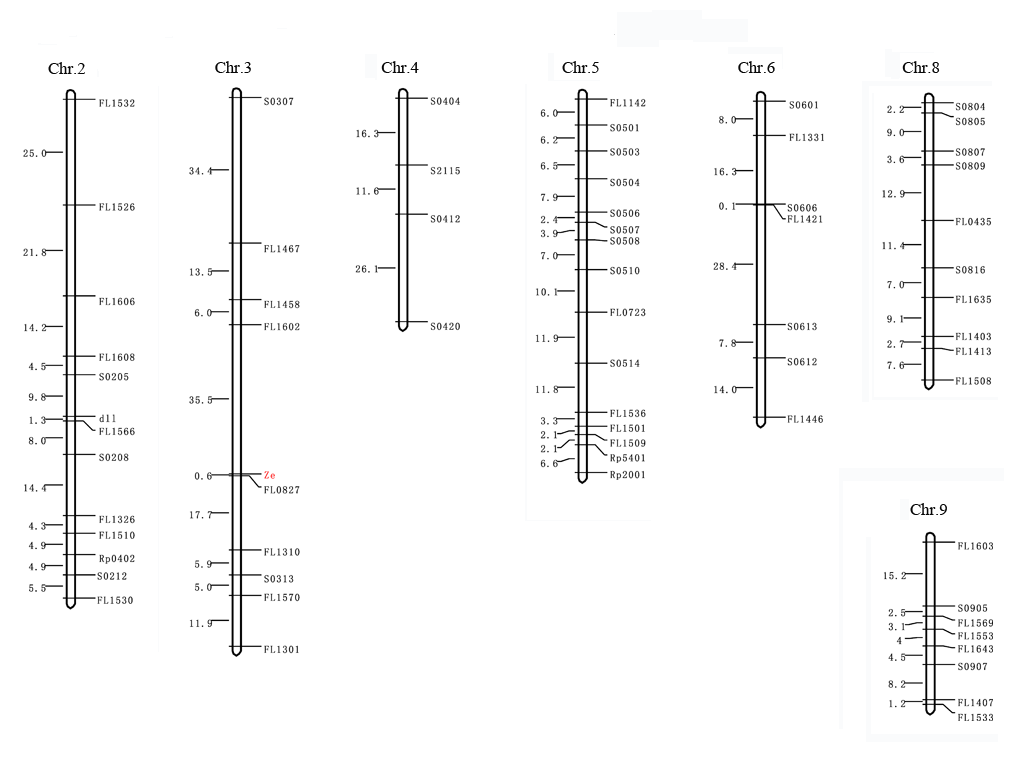

Supplement: Additional file 1 — NF linkage maps, part I. The most likely linkage maps for seven chromosomes (2, 3, 4, 5, 6, 8 and 9) generated by the NF data set are shown. [file 1471-2164-10-389-S1.tiff]

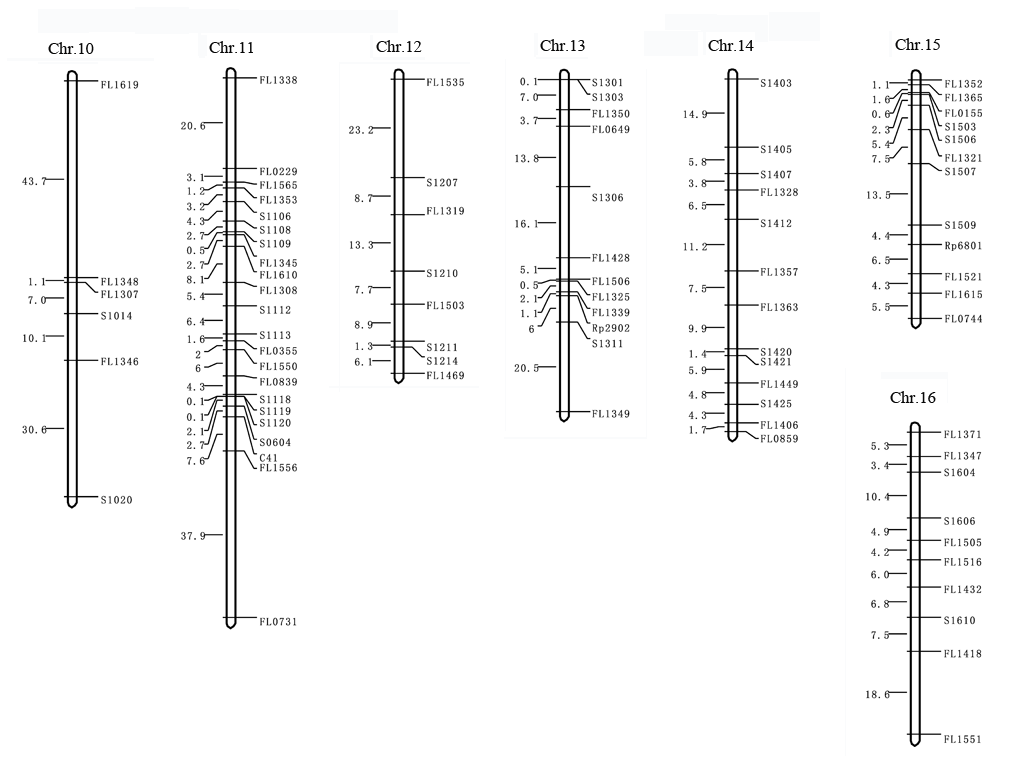

Supplement: Additional file 2 — NF linkage maps, part II. The most likely linkage maps for seven chromosomes (10, 11, 12, 13, 14, 15 and 16) generated by the NF data set are shown. [file 1471-2164-10-389-S2.tiff]

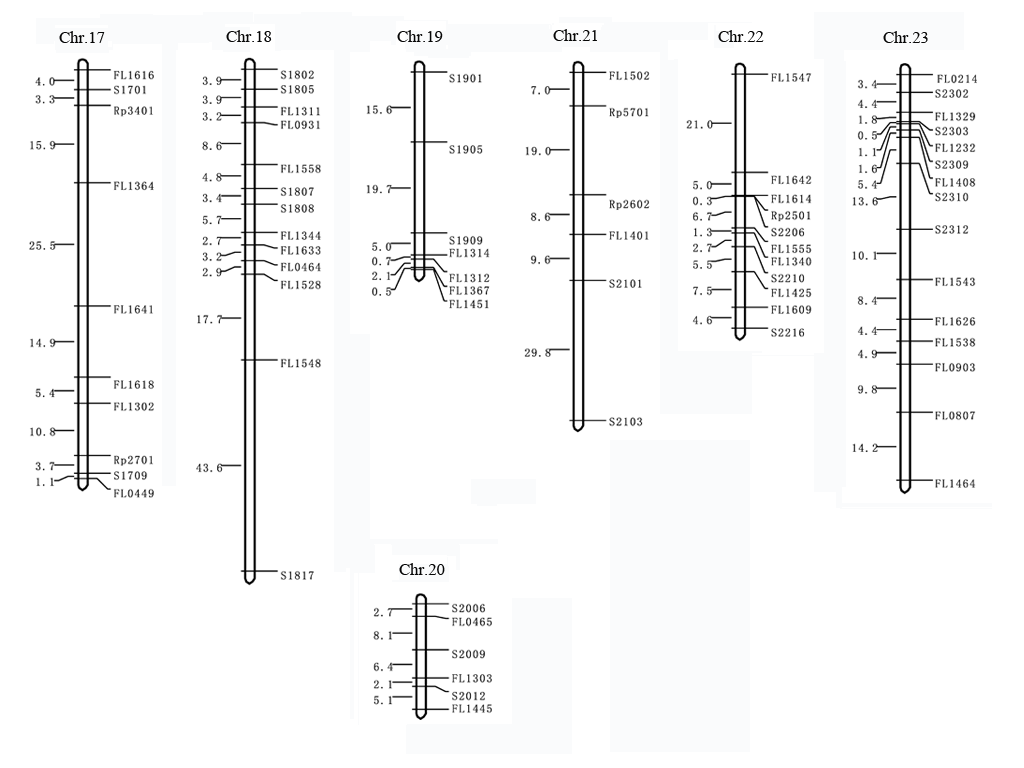

Supplement: Additional file 3 — NF linkage maps, part III. The most likely linkage maps for seven chromosomes (17, 18, 19, 20, 21, 22 and 23) generated by the NF data set are shown. [file 1471-2164-10-389-S3.tiff]

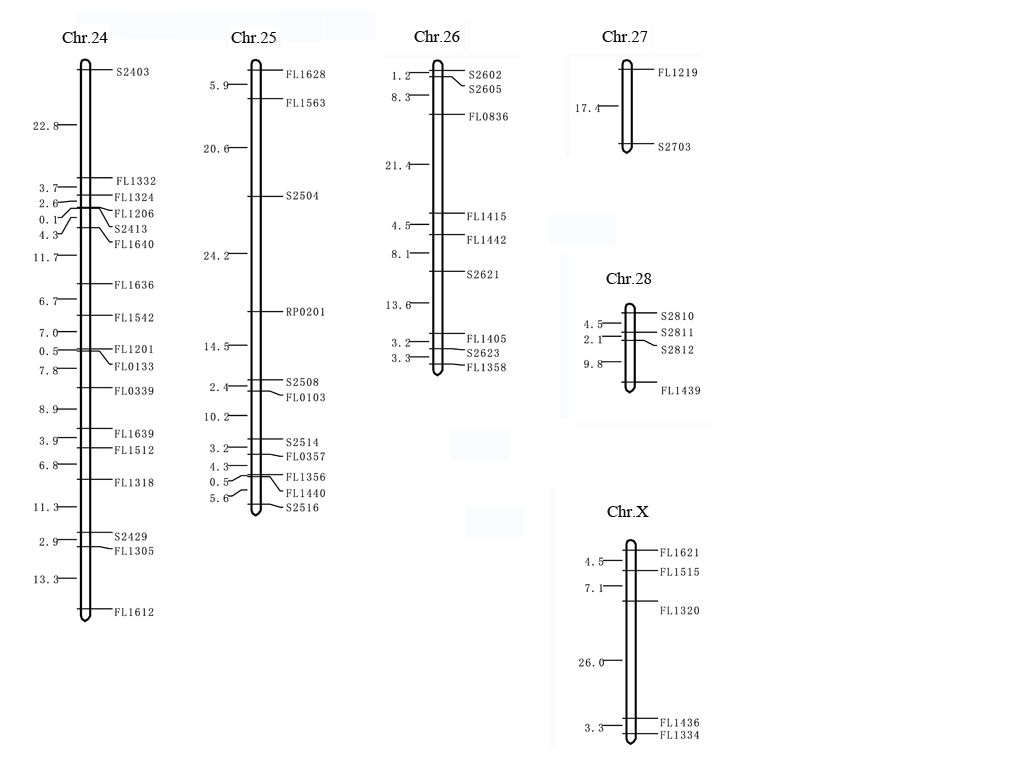

Supplement: Additional file 4 — NF linkage maps, part IV. The most likely linkage maps for six chromosomes (24, 25, 26, 27, 28 and the unassigned group X) generated by the NF data set are shown. [file 1471-2164-10-389-S4.tiff]

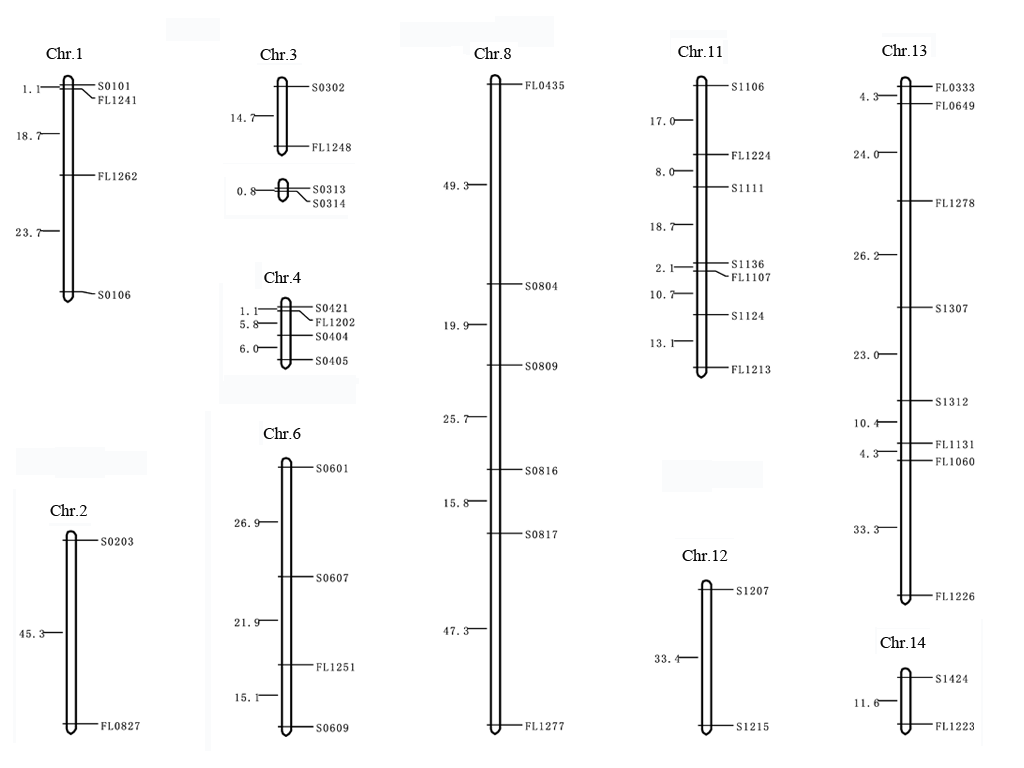

Supplement: Additional file 5 — JL linkage maps, part I. The most likely linkage maps for ten chromosomes (1, 2, 3, 4, 6, 8, 11, 12, 13 and 14) generated by the JL data set are shown. [file 1471-2164-10-389-S5.tiff]

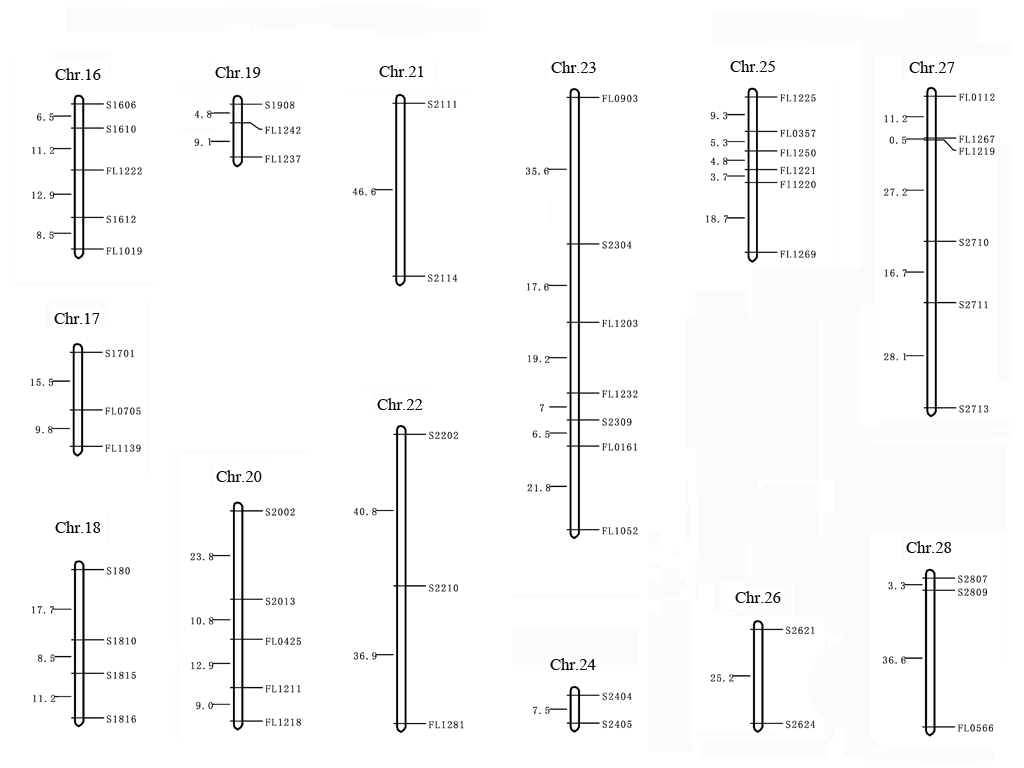

Supplement: Additional file 6 — JL linkage maps, part II. The most likely linkage maps for 13 chromosomes (16, 17, 18, 19, 20, 21, 22, 23, 24, 25, 26, 27 and 28) generated by the JL data set are shown. [file 1471-2164-10-389-S6.tiff]

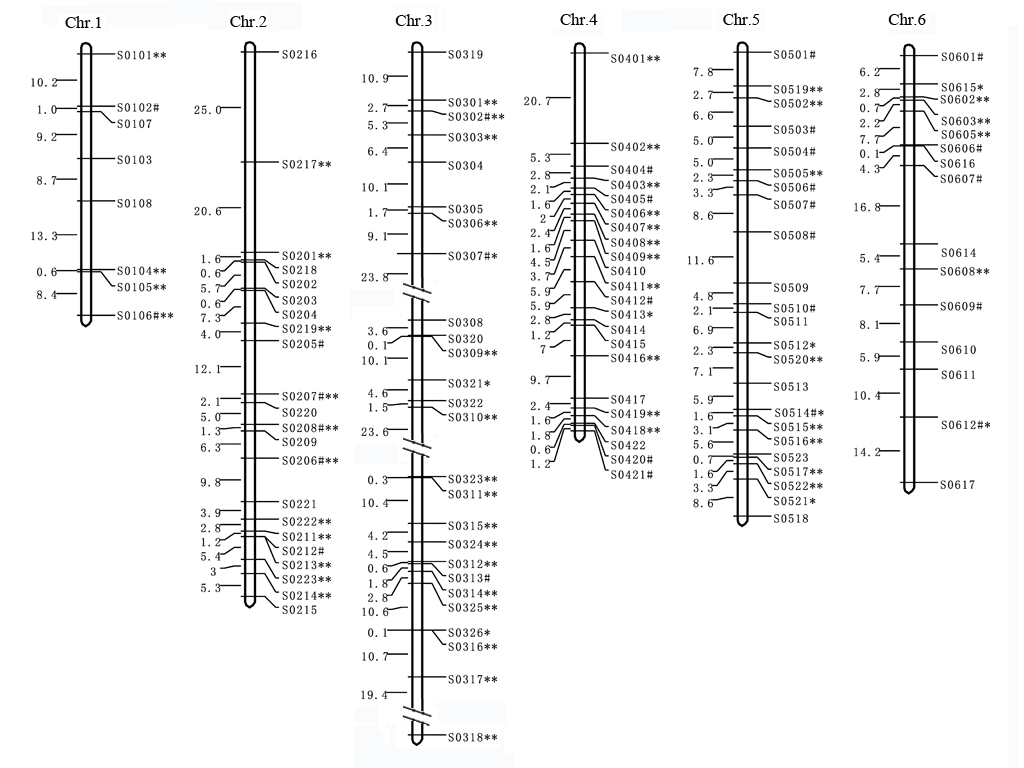

Supplement: Additional file 8 — Integrated maps of three mapping populations, part I. The most likely linkage maps for the chromosomes integrated with three data sets are shown in this file and the following four additional files (additional file 9, 10, 11 and 12). We removed five loci that showed significant disagreement in the distance to adjacent loci (S1401), position in the group (S1116 and S2711), or grouping result (S0210 and S1140). Furthermore, a number of markers with improved likelihood over the initial map appeared in seven groups: Chr. 2 (S0206–S0209; Figure 2B), Chr. 3 (S0312–S0315), Chr. 4 (S0403–S0404 and S0418–S0419), Chr. 9 (S0901–S0907), Chr. 18 (S1813–S1815), Chr. 20 (S2018–S2019), and Chr. 26 (S2617–S2622). Inverted orders were observed in Chr. 22 (S2209–S2210 and S2211–S2212) and Chr. 10 (S1012–S1013) because the CAPS marker was not included. The sites that contained confirmed neighboring markers are indicated by a star. An additional star indicates that a polymorphism was detected. Chromosomes 11' and 25' shared one common marker; however, their order could not be determined. This file contains the maps of six chromosomes (1, 2, 3, 4, 5 and 6). [file 1471-2164-10-389-S8.tiff]

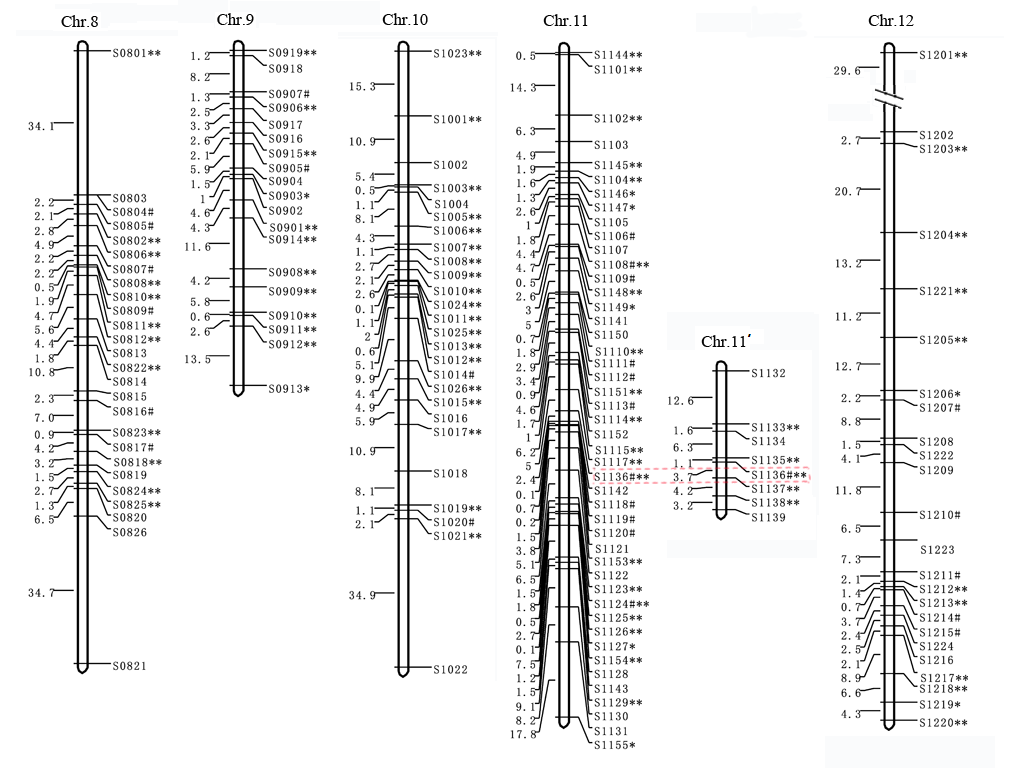

Supplement: Additional file 9 — Integrated maps of three mapping populations, part II. The most likely linkage maps for five chromosomes (8, 9, 10, 11 and 12) integrated with three data sets are shown. The sites that contained confirmed neighboring markers are indicated by a star. An additional star indicates that a polymorphism was detected. Chromosome 11 and 11' shared one common marker; however, their order could not be determined. The map of chromosome 7 is not included here because there were not any new identified markers on it. [file 1471-2164-10-389-S9.tiff]

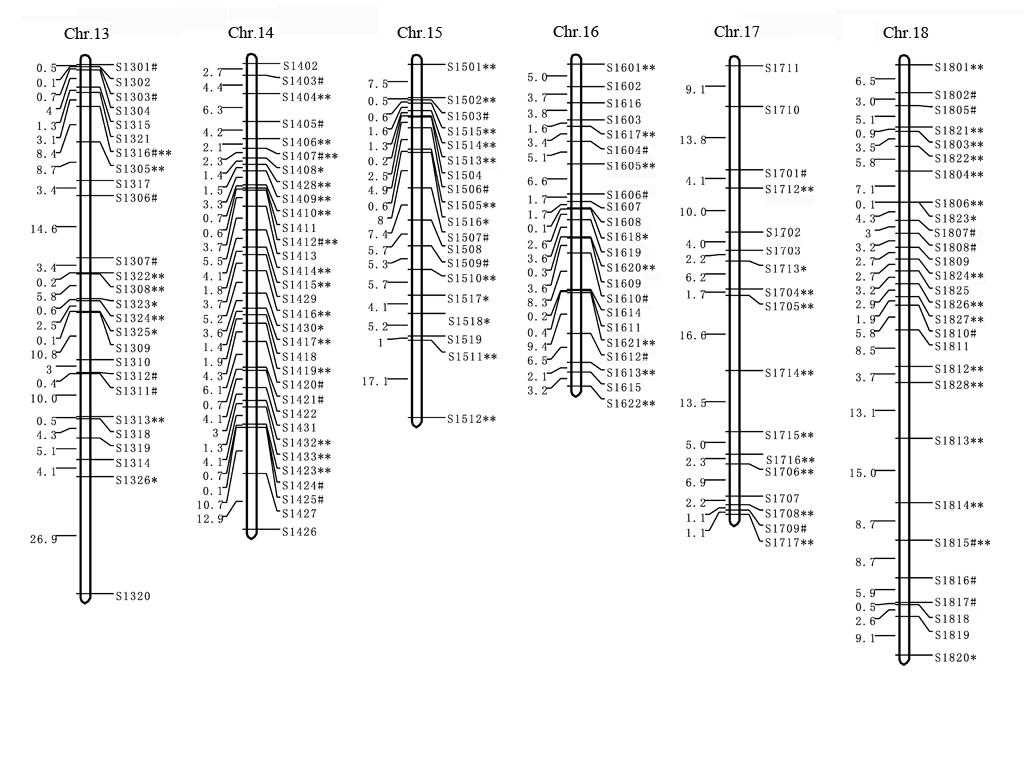

Supplement: Additional file 10 — Integrated maps of three mapping populations, part III. The most likely linkage maps for six chromosomes (13, 14, 15, 16, 17 and 18) integrated with three data sets are shown. The sites that contained confirmed neighboring markers are indicated by a star. An additional star indicates that a polymorphism was detected. [file 1471-2164-10-389-S10.tiff]

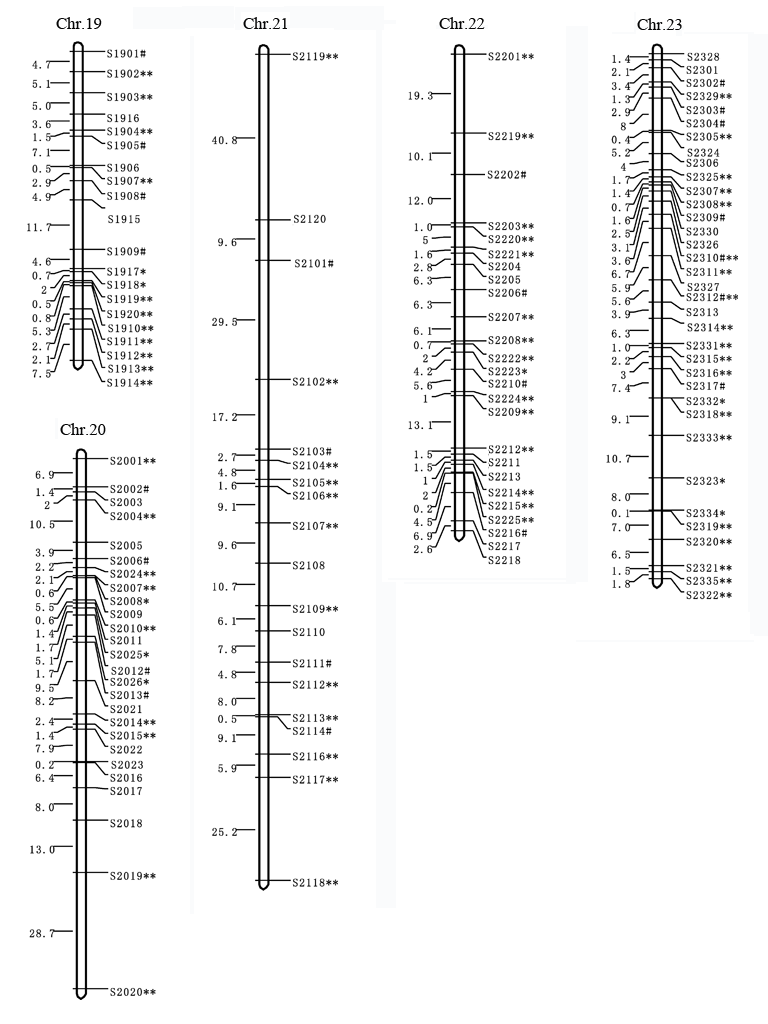

Supplement: Additional file 11 — Integrated maps of three mapping populations, part IV. The most likely linkage maps for five chromosomes (19, 20, 21, 22 and 23) integrated with three data sets are shown. The sites that contained confirmed neighboring markers are indicated by a star. An additional star indicates that a polymorphism was detected. [file 1471-2164-10-389-S11.tiff]

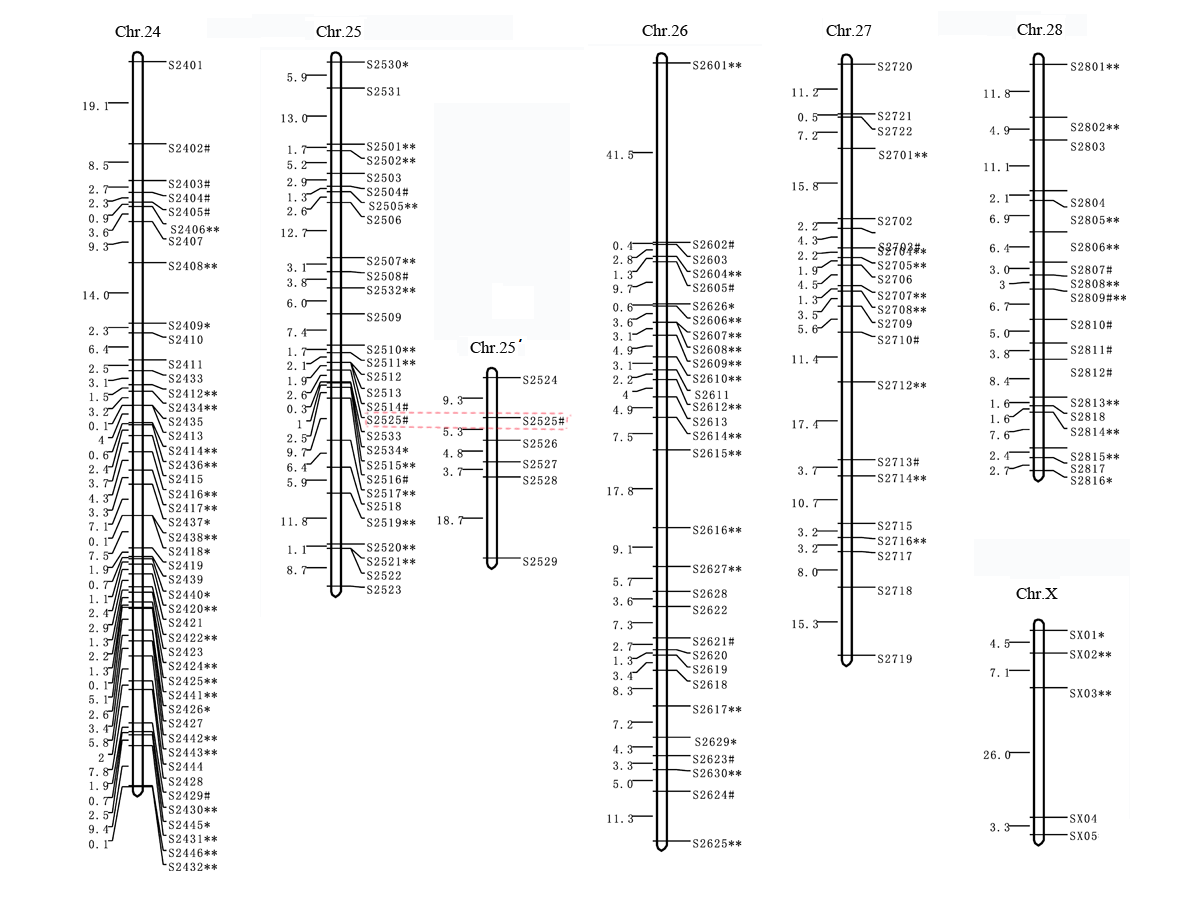

Supplement: Additional file 12 — Integrated maps of three mapping populations, part V. The most likely linkage maps for six chromosomes (24, 25, 26, 27, 28 and the unassigned group X) integrated with three data sets are shown. The sites that contained confirmed neighboring markers are indicated by a star. An additional star indicates that a polymorphism was detected. Chromosome 25 and 25' shared one common marker; however, their order could not be determined. [file 1471-2164-10-389-S12.tiff]
