# Supplementary material for: An integrated genetic linkage map for silkworms with three parental combinations and its application to the mapping of single genes and QTL
Source: BMC Genomics. 2009 Aug 21;10:389. doi: 10.1186/1471-2164-10-389 (PMC2741490; doi:10.1186/1471-2164-10-389)
Supplement: Additional file 7 — Information on the markers used in this study. This table contains the work name, map name, forward primer sequence, reverse primer sequence, labeled type, and population source. [file 1471-2164-10-389-S7.doc]

| Work  name | Map name | Forward primer sequence | Reverse primer sequence | Labeled type | Marker source |
| --- | --- | --- | --- | --- | --- |
| Markers for map construction | | | | | |
| FL0101 | S1807 | TTTATTTGTTAGGCTCGTTACTGTCA | CAAAAGCACAGCTACTTCCGC | FAM | from pervious DC |
| FL0103 | S2532 | GCGTCGGCGAACTTATTG | TGTCCAGTAGTAAGTGTATGCCAACC | FAM | NF generated |
| FL0112 | S2720 | GGCAAAGACTGGATGTGAGATGTAA | ATATTCAGCACAGCGTTTCCTTAA | FAM | JL generated |
| FL0122 | S1118 | ACGGCAGCACACGCATAAAAC | GCACTAGTGTCACGACGGCAATC | FAM | from pervious DC |
| FL0124 | S1506 | TTGCGTTGTTGATGTCTATGGG | AGCGAGGAGACGGGAATAAGA | FAM | from pervious DC |
| FL0126 | S0805 | AACCTTTATTTATCATTTGAGTAAGTCCA | TCCTTTACTGAAACGATAATAGCCAA | FAM | from pervious DC |
| FL0130 | S0905 | GTTCATCAAATCATACTATACCAAATCAAT | ATCGGCTCAACTTGTGGGAA | FAM | from pervious DC |
| FL0133 | S2440 | CGTAAAACGCCACAAGTCCTAAT | GGGATGCGTTGCTGGCTTC | TET | NF generated |
| FL0134 | S1909 | CAGTGGCGGACTTAATGGCT | AATTCCCGTCATTAATATTTATCCAA | TET | from pervious DC |
| FL0141 | S2309 | TAGGTACAACAGGAATGCCCG | ATTGTGAAATGGCACTTACTGTTGT | TET | from pervious DC |
| FL0142 | S2115 | CGGGTTCTTCGTTTGTGAGG | GGTGACCAATGCTTGAGGGG | TET | from pervious DC |
| FL0145 | S2812 | AAAATTAGACGACATGCTTAACCTCA | CACCTACCCGTGCGGACTCT | TET | from pervious DC |
| FL0155 | S1515 | CGAAAACGGTTAATAATCGTGAATG | TGGTGAGTGGTTACCGTCGC | TET | NF generated |
| FL0157 | S1610 | CATCCCCGATTGAGCCTTGC | ACTGATCCCTATACAAGCCCTACC | TET | from pervious DC |
| FL0158 | S2312 | CCGCGGACACTTCAAAACT | AACCCCACTGAACGGACCC | TET | from pervious DC |
| FL0161 | S2326 | CGGGGACGGTGCTCCTTAC | TGCCCACGGTCTCCTCG | TET | JL generated |
| FL0163 | S1901 | CCAAACGATAAGCACTATTCCCG | CAAACGATCTTGATACTTTACCCGA | TET | from pervious DC |
| FL0164 | S0507 | ACATCAGGCAGACGCTCGGT | GGTAGTGCAGAGTAGATAGTGGTAGGTG | TET | from pervious DC |
| FL0173 | S2101 | CATTAGTGTTCGTCGCCAGTCAA | TTACGTCCGCGTTTTGGCT | HEX | from pervious DC |
| FL0176 | S0404 | CGTTTGCCTACGCTTGCC | CACGCAGAGGCAGTTGTCG | HEX | from pervious DC |
| FL0214 | S2328 | ACAGGGCGTTCGTCCTTTGC | CCGTGAGATTTAGGACAGCGATA | FAM | NF generated |
| FL0229 | S1145 | GACGAAAGGTTGTGGTAGACAGTTGA | CTTCGTGTCTCAAAATGTGGTCA | FAM | NF generated |
| FL0304 | S1503 | ACTAATGAACAAATCAAATAGAAAACGA | CATCGCCTGTGAATGAACGC | FAM | from pervious DC |
| FL0305 | S1108 | TTGAAGATAGAGCGAAGTGGAGG | GCGGCAGAAATAGGACGGT | FAM | from pervious DC |
| FL0306 | S0508 | GGGTGTTGTAGATGCTGGCG | ACACTCAGCATTGGGTGGTCA | FAM | from pervious DC |
| FL0308 | S1425 | GCGACGCACTCCATCAAGC | TGCTTTCGTCCTTTCTTATCCCT | FAM | from pervious DC |
| FL0310 | S1211 | CTGGGATGTTTAGTGTTAAGTGCG | GCGATAAGACCGCCAATTGT | FAM | from pervious DC |
| FL0313 | S2623 | TGACTGTGGGCTGATAAGCATAA | TCTTGTCCACCTGATGATTGTCTG | HEX | from pervious DC |
| FL0329 | S0816 | GAAATCCGTTTGAAGAATCCACA | CATCCGTTGAATGAGTATCGTTTG | TAMRA | from pervious DC |
| FL0333 | S1315 | GAAGACAGAGCGAAGTGGAGGA | CAAGGAGTGTTTGATCGAAAGTGT | FAM | JL generated |
| FL0336 | S2006 | CGTATTTTAGTGTATGACTCGGATGA | CGCAAAACAATCAAGTGGTCC | FAM | from pervious DC |
| FL0339 | S2441 | TCTGCCAGCCGTTATGTAGATTAG | CCCCTTTGTGGCTTTTCGTTTA | FAM | NF generated |
| FL0346 | S2009 | AGGGCGGATTGGTAGGTGAG | TGAAATGTCACCCCTCGATCTAA | HEX | from pervious DC |
| FL0347 | S2621 | GCCAAATGATTATTGTCAGCCTG | GGATGGACGGAAAATCGCAG | HEX | from pervious DC |
| FL0355 | S1151 | TCAAGACTGCACTATACACAATTAAACAA | AGTGAAGGAGTGCAGAATGCTAATAT | TAMRA | NF generated |
| FL0357 | S2525 | AATGAGCTGTCTTGCTTAGAAAACTTAT | ACCCTTAGCAGTCGAGTACAATTTT | TAMRA | JL generated |
| FL0357 | S2525 | AATGAGCTGTCTTGCTTAGAAAACTTAT | ACCCTTAGCAGTCGAGTACAATTTT | TAMRA | NF generated |
| FL0364 | S0504 | AGGACTTAGAAGCTACGATGGAATA | CCTGCCTGTTATCTGTTTTGAATG | FAM | from pervious DC |
| FL0366 | S2206 | GGGTTAGAGGTCCCAAACGATG | CAGGTCACTTAGTCTTAACATTGCG | FAM | from pervious DC |
| FL0374 | S1210 | CACAGATTTCGCCAAGACTACACTAT | TTACGAAAACGGATGGAGACG | HEX | from pervious DC |
| FL0386 | S0606 | TCAAGAAACCCAAGCATCCAAC | TTAGCGATAAGACCGCCTATTGTA | TAMRA | from pervious DC |
| FL0401 | S0604 | TTGGCAAGTGGCAACACAGAT | ACTGAACCGATGCCTAAATACCA | FAM | from pervious DC |
| FL0404 | S1606 | AAATAGAAACTGACGATGAGGTAATAGC | ACAGATTGATTCCAGAAACGAGC | FAM | from pervious DC |
| FL0407 | S0809 | AACATTTGCTTAGGACTGAATTTACAC | AATAATAACTTTTACACGCACCTACACTT | FAM | from pervious DC |
| FL0409 | S2429 | AGATAACAAACCGATAACGGAGATT | GGTTCTCCTTACCTCTACCCTCCAT | FAM | from pervious DC |
| FL0410 | S1421 | CAAGGTGGAAGGATGTAGTGCTG | GTCAGCGTCGTAGTCTCGTTTCT | FAM | from pervious DC |
| FL0416 | S1119 | AAAACACGGTGGTGCAACGC | CTACAAAATACCATTACTGACACCGA | FAM | from pervious DC |
| FL0418 | S0612 | TCCCACCGACATCTGCATATT | CGGGGACGACACGCACA | FAM | from pervious DC |
| FL0419 | S1106 | GCCGTGCTGGACATAGGACTT | CCAGTCATCTTGGAATGCGTTT | FAM | from pervious DC |
| FL0421 | S1604 | ACAGCATCCAGGTCCGTTCC | GCCGAGTAAAGTATTTGCGTCAT | FAM | from pervious DC |
| FL0423 | S2811 | CGATGGAGACAGTTGGTTGACG | CCTTGCGTCGTCCGTCG | FAM | from pervious DC |
| FL0425 | S2021 | CAGGTTATTCTGTTTCAAGACGCAT | AAATATGCTCCGAGGCGTGA | HEX | JL generated |
| FL0426 | S2303 | GCTAAAAGGCGACATCTGCG | TTAGGTGCAAATGCTTGTGACTCT | HEX | from pervious DC |
| FL0429 | S1709 | AAGGGATTCTCTACCAGTCAACCA | TTTGACGCTGGCTTATAAATACTGTAT | HEX | from pervious DC |
| FL0435 | S0822 | CCAAATGATTTCTTGTCCACCTG | CGAGAATCTTTGTGGGGCTGAG | HEX | JL generated |
| FL0435 | S0822 | CCAAATGATTTCTTGTCCACCTG | CGAGAATCTTTGTGGGGCTGAG | HEX | NF generated |
| FL0437 | S0412 | CGTTTCCAAATGATTCTTATCCACC | GTGGAAGAGTTACGACGGCAA | HEX | from pervious DC |
| FL0442 | S2504 | AAAAGGTCGTCAGCCAACACTAA | AATAAGGACATATTGTATTGTTACGCC | HEX | from pervious DC |
| FL0443 | S1303 | GCATCCCCACTATCGTCAACTAAAC | TCCGTTCAGGCTTATCGTCAGTA | HEX | from pervious DC |
| FL0447 | S0212 | TGTGATGAAAATGGTAGAGCAAGG | GCTGTCCCTGCAAAACGAATA | HEX | from pervious DC |
| FL0449 | S1717 | GACACCCCGACGCAGACAT | TTTTGCGGGTTTGATTTTATTACA | TAMRA | NF generated |
| FL0456 | S1412 | CGTGTTTTCTCCATCCCCAA | GTAAATTAAACGGTTGTTTGGAAGAG | TAMRA | from pervious DC |
| FL0460 | S2508 | CTTCGCATATCATAATAGGCTTTTG | GGTTTTACGGGATTCCCTCAGT | TAMRA | from pervious DC |
| FL0464 | S1826 | AAATGCAAACATCCGTTATCTAC | AATGGCAGCCCGTCAACC | TAMRA | NF generated |
| FL0465 | S2024 | AACTGCTTTTACTGGTAGGTTGGTG | CGGACATCGGTGGAAGCATA | TAMRA | NF generated |
| FL0469 | S2514 | AAACATCAAACAGTTAGGTGGGC | ACTGGTTTCGCATTGCTTGTG | TAMRA | from pervious DC |
| FL0506 | S2302 | CTCACAGGACAAGCTCCAAAG | TCCATAGTTCGGGAGCTGAGG | FAM | from pervious DC |
| FL0509 | S0807 | TGAACTATGCTATTCCATTGTCGTG | AAATGTCAATATGAAACAAAAGAGTAAATC | FAM | from pervious DC |
| FL0522 | S2602 | ACATCAACTACAGAAAAGGAGGGAA | TGCTCCTTACTCCTACCCTCCAT | FAM | from pervious DC |
| FL0536 | S1507 | TGCCCCACCCTTCAAACC | GCGTAGCCACTGAAGGTTTACTT | HEX | from pervious DC |
| FL0552 | S0208 | CCACGGCCAAGGCGAGA | CGTGGTGAGCAATACGTCCTG | TAMRA | from pervious DC |
| FL0557 | S2210 | TTGGTGTTACAATGTGATGCCC | AGGTTGTTGTTTTCAGATAAGTTTGC | TAMRA | from pervious DC |
| FL0560 | S1407 | CAAAACCCACTTAATACCAAACGG | AGAAGAATCGGCGAAAGAAGC | TAMRA | from pervious DC |
| FL0563 | S0601 | CCTAAGCTAAAGGAGTCCTAATCACA | GATGGTGAGTGGTTAGGGACGT | TAMRA | from pervious DC |
| FL0566 | S2817 | TTAGCGATAAGACCGCCTATTGTA | TCAAAACCTTCCAAGTGAATCCTC | TAMRA | JL generated |
| FL0572 | S2703 | CCTCCCGTTTGCCTTGACA | GCTTGAATGCGTGATGCGG | TAMRA | from pervious DC |
| FL0639 | S1808 | CCGAGTAGAACTGCGCTAATGTACTT | GGGTCTGCGACGGCGAT | HEX | from pervious DC |
| FL0649 | S1316 | TGCCTTGTAGGTAGACGATGGAG | AATTAGCGTAAAGACCAGGGCA | TAMRA | JL generated |
| FL0649 | S1316 | TGCCTTGTAGGTAGACGATGGAG | AATTAGCGTAAAGACCAGGGCA | TAMRA | NF generated |
| FL0660 | S1014 | CGTCATTTCGTTTTGAGGATTAGA | CAATATCCAGCATGGGCACAA | TAMRA | from pervious DC |
| FL0667 | S0313 | CAGGATAGTGGATGATTCTTTTCG | CCTATTGTCCAAATGTATGTGCTTTT | TAMRA | from pervious DC |
| FL0669 | S0514 | CAGGCTTTGGATGATGATAAATACA | GAGGATGTTGTGCGGACGC | TAMRA | from pervious DC |
| FL0672 | S0501 | TTACCGACACTCCTGGACATCA | CGCATTCATCCTTATTGTATCGTAG | TAMRA | from pervious DC |
| FL0705 | S1710 | GGGATAAGTGGGTCGTTTTGATT | TGAGACCCAATAATGTCCCGAG | FAM | JL generated |
| FL0713 | S0503 | TCATTTCATTCCCACTGCCTTT | CGGTAGGCTGTAAGCTCGTCA | FAM | from pervious DC |
| FL0721 | S1113 | GGAGTAAAATCGCCGCCATA | AACTCCTTCGACATCCTCCGTAC | FAM | from pervious DC |
| FL0723 | S0520 | CTGCTGCTACGAGCCCGA | CGCTTAATTCGTTTCCAAATGA | FAM | NF generated |
| FL0724 | S1301 | GCTCAACGGGGAAGGTCACC | TGGAAGGATGTAGTGCTGAAGGAC | FAM | from pervious DC |
| FL0728 | S1509 | GTGGTTATTGGGAACTTTTATGATGT | CGTATTAGGGAAGAAAAGATGGCT | HEX | from pervious DC |
| FL0735 | S2216 | CGCATTGATAGAACGCTGGC | CTTAGCGATAAAACCGCCAATT | HEX | from pervious DC |
| FL0742 | S1214 | AATGGATGCGTGCCAGTTTG | CGTTAGTTGCTCTACGCTTTTGG | HEX | from pervious DC |
| FL0744 | S1519 | CGACGGCAACAATAAGGCAAT | AACTGTCCCAAAGTGTTCCAAAA | HEX | NF generated |
| FL0747 | S1311 | ACTACCCGTAGTTGTAATTTGATCTTTT | ACGTGTTTGGTTTGCTCGCT | HEX | from pervious DC |
| FL0748 | S2012 | CGGCAGCCCCGTGGATT | CGACTTGTGAGAATCAAAGAAACTG | HEX | from pervious DC |
| FL0756 | S1112 | TATGCCAAGGTGTTCGACGG | AATTTGCTCATAAACCCGCG | TAMRA | from pervious DC |
| FL0758 | S0420 | TGAACACAAAAATCATTAAAGGAACA | ACGGAAGACCAGAAATCTCGTAA | TAMRA | from pervious DC |
| FL0761 | S1207 | CTCAGTTGGTCGCATTTCCG | TCCGTGGAATTTTATTTCAGTTGTA | TAMRA | from pervious DC |
| FL0762 | S0804 | TGTGCTGCGTGGATGTAGGAG | GACGGGCAGGCGGCT | TAMRA | from pervious DC |
| FL0775 | S2310 | GGCGCTCAACCAACTTACACCT | TCGAGGCCGCTGTGATAATAG | TAMRA | from pervious DC |
| FL0805 | S2304 | ATGACAATACGCTTTATCACCAGG | ATGGGTTAGTTCGCTCGTCG | FAM | from pervious DC |
| FL0807 | S2334 | TGCCGTTTGCGCCCATA | TTATGAAAACCGCGTAGTTAAATTG | FAM | NF generated |
| FL0810 | S1405 | GCATTCTTGTCCACCTGAGGG | GCTGAGGGTCGTGGGTTCG | FAM | from pervious DC |
| FL0815 | S0205 | TCTTGACAGGATTGAAATGAGGC | GCGAGATATGGATTATTTAATGGCT | FAM | from pervious DC |
| FL0818 | S2403 | TTCCTCGCCCTCGCTTACAC | CTAAGTCCATCTTCTATTGACAGCGT | FAM | from pervious DC |
| FL0821 | S2810 | GCACTGCTCTTGCTGGGGT | CGGTTCCTCCGTGCCAA | FAM | from pervious DC |
| FL0827 | S0323 | CACCGTCGGCGGCACA | TTTTAGTGATAAGACCGCCTGTTG | HEX | NF generated |
| FL0829 | S1120 | ACATATAAAACACGGTGGTACAACG | CGGCAGCACACGCATAAAACTA | HEX | from pervious DC |
| FL0831 | S0510 | TTTTGCGGGATAAACCGATACT | TATTTTAATGTCAATTGCGTTTTAGTG | HEX | from pervious DC |
| FL0836 | S2626 | ATAGCGTTTTGCGAGTTTGATTT | CGGAGGTAATGAGTGAAGCGG | HEX | NF generated |
| FL0839 | S1153 | TGAGTCTGTCTATCCATCCTACG | GTTGGGGTGTCTCATTCATTTGT | HEX | NF generated |
| FL0841 | S1403 | CTTCACTGCTCGCGTTCCC | GAGGCTTTCGTGCCAATGAG | HEX | from pervious DC |
| FL0845 | S1802 | TTTGAAGAGCAGGTCAGCCG | CGGGATCGATGGAAACAGCT | TAMRA | from pervious DC |
| FL0850 | S1420 | CCCTTTGCCATTTTGTCACTTC | TTGTCTACCTGATTGTTGTCTGGAA | TAMRA | from pervious DC |
| FL0852 | S1805 | GCAAACAAAAGTTAGTTACGGATGA | AATCGCTTGTTGACTGTTTTCTGA | TAMRA | from pervious DC |
| FL0859 | S1433 | CCTCGGAATTGAAGGACGATAT | ATGGTAGTGCAAGGTAGAGGGG | TAMRA | NF generated |
| FL0865 | S1306 | GGAAACAAAAGAAAGCTAAAATACACAT | TTGACCAGACTATAAGCAATAAGAAAAGT | TAMRA | from pervious DC |
| FL0903 | S2323 | GCCTCTGTTTTATTCAACTCACGC | GGTTTTCTGATTTATTGTTCTATGGTATTA | FAM | JL generated |
| FL0903 | S2323 | GCCTCTGTTTTATTCAACTCACGC | GGTTTTCTGATTTATTGTTCTATGGTATTA | FAM | NF generated |
| FL0912 | S1905 | CTTGCTCCATAAGGGGAACATT | ATGCGACGGATGCCTGAA | FAM | from pervious DC |
| FL0918 | S0907 | GCGGGCGGAGCTTGTACT | CGCATTGTTTATTTTGTTGGGG | FAM | from pervious DC |
| FL0931 | S1822 | GCAAGAACCGTGCCTCGC | AAAAGGTTAAGTCGTGAACAGATGC | HEX | NF generated |
| FL0947 | S1109 | CAAATATCCGTAAAGTCCCCAG | CGACCCTACCTAAAGTGGGATAAGA | TAMRA | from pervious DC |
| FL0948 | S2413 | GCTCATCCACCCATCTAAGCAATA | GGCCCCGGTCGTTGAGG | TAMRA | from pervious DC |
| FL1009 | S0613 | CATTGGGAACGGTGGCTTG | CGCTTTTGGCGATAATGTACTTTT | FAM | from pervious DC |
| FL1019 | S1615 | CACCTAGTTTTACTTCACGGACCAT | CCCTTGGGCTTAAGTCGGTTT | FAM | JL generated |
| FL1051 | S2516 | GCCACCCACTGTTTCCTACG | TGTAAATTAAACGGTTGTCTGGAAG | TAMRA | from pervious DC |
| FL1052 | S2327 | GGAACGCTGAATCCACGGA | TTTAATGTATCGCACTGTTTATTTGTTT | TAMRA | JL generated |
| FL1060 | S1319 | GAGACTTTGTGTCGTGGGTCCTAC | CCTTCAAACCGAAACGCATTACT | TAMRA | JL generated |
| FL1102 | S1020 | GGCTGACTGCGAACAAACG | CAATAAATCTGTGAAGGAGTTGGTGA | FAM | from pervious DC |
| FL1107 | S1142 | TCTGTGAACTAATCCCGTGCATA | GGGAGAAGGGTAGAGGAATGCT | FAM | JL generated |
| FL1108 | S0307 | GCTTGTGACGTACTTTCGGACTT | CCGAACTGAATCAGCAAGAAACTC | FAM | from pervious DC |
| FL1124 | S1701 | GTCATTGGGAGTTTGAAGTTTCG | ACGGGCTTTCTTTGCTAGATGT | HEX | from pervious DC |
| FL1131 | S1318 | ACATCCGCCGAAACCACTGA | GCAATATTCGAGTTAATGAAAGCCA | HEX | JL generated |
| FL1132 | S2605 | CGGCGATAGATGGCGTTACTC | TTAGGCCACTTTTGTGCTGATG | HEX | from pervious DC |
| FL1139 | S1711 | CGGCACTTAAAAGTTTTCATATCAATC | CTGACAGTGGTGAGTTAATAAAACAAAA | HEX | JL generated |
| FL1142 | S0519 | CGTGATTCGAAACGTCAAGTACATAA | TGGCTCATTCGTGGCTCATT | HEX | NF generated |
| FL1152 | S0506 | GCAAAGGGAACAATGGTGGGT | TTAGTTTGTTATTAACGGATGTCGC | TAMRA | from pervious DC |
| FL1201 | S2439 | CCGACGGTCAAAATTCTGCG | CCGTCATTGACCATTAGTAAGCC | FAM | NF generated |
| FL1202 | S0422 | CCGTACTGGGAGCACAAGC | CGGGTGGAGTTGCTCGGTA | FAM | JL generated |
| FL1203 | S2324 | CGTTTATACCATCGCACCGC | TTATGGGTACGCATACAGTCCAA | FAM | JL generated |
| FL1206 | S2435 | CTGCTCACAATCACAACACACGT | TCACGATTGACTTCCACGCTG | FAM | NF generated |
| FL1211 | S2022 | AGCAGTGCTTAAGTGAACCTACCA | GCATTTGCGGATTTCACTGG | FAM | JL generated |
| FL1213 | S1143 | TCTACCACAGCCAAACGATATCA | AAATTTCATACTCCTCCGTCCG | FAM | JL generated |
| FL1218 | S2023 | GCGGTTTCCTCCCAAGACT | GGATGTATCAAACGCAAAGCAG | FAM | JL generated |
| FL1219 | S2722 | AGGCAGGCAATACTAACTCTTCTTC | CGGACACGGGAGAATAATCG | FAM | JL generated |
| FL1219 | S2722 | AGGCAGGCAATACTAACTCTTCTTC | CGGACACGGGAGAATAATCG | FAM | NF generated |
| Fl1220 | S2528 | GTTTATGGTGTTTCTCGTAGCCC | GGTTCCGGTTGTCGCTCG | FAM | JL generated |
| FL1221 | S2527 | GTTTGCCTCGGCTGCCATAA | CGCCAAACAAGAATATTTACCCG | FAM | JL generated |
| FL1222 | S1614 | AAAATCGAGAAAACCAACGTCC | AGAAGCAGAGCCATCTTAAATCG | FAM | JL generated |
| FL1223 | S1427 | TCTACGTTGGTGTCCTGTCATTATTC | AAAATTTGTGCTTCCGTGCG | FAM | JL generated |
| FL1224 | S1141 | GGAACAAGGGAAGTTTTATTTTATGC | GATGCGGGTATTTCCTTATTTGA | FAM | JL generated |
| FL1225 | S2524 | ACGGCAGAAATAGGCGGG | CGCTTTTAGTGATAAGACCGCATA | FAM | JL generated |
| FL1226 | S1320 | CGAGGAGAAAACAGACGAATAGACG | AAATATGCTTCCCGGTTGACTG | FAM | JL generated |
| FL1232 | S2325 | ACGCACTGAAACGGGCATT | GTTAGCATCTGCATAGGCGTGA | HEX | JL generated |
| FL1232 | S2325 | ACGCACTGAAACGGGCATT | GTTAGCATCTGCATAGGCGTGA | HEX | NF generated |
| FL1237 | S1916 | GATTTCGCCTCAGGGGTTG | TCCGGTCGTAGATGGTGAGAA | HEX | JL generated |
| FL1241 | S0107 | CCCTATCCCTAATGATTCTTGTCCA | CGATGCTCGATCTGATTTACCA | HEX | JL generated |
| FL1242 | S1915 | GCCGCTGCTAACCGAAAGA | TATGATCTTATCTTTTCAGAATTTGGG | HEX | JL generated |
| FL1248 | S0319 | ACGAGTGGTTCAAGTGAGGAGC | AAAAGAATGGGAAATGAATGGC | HEX | JL generated |
| FL1250 | S2526 | TCCCTTGACGCCGAGACAC | CGATCGCTTCACAGGCACA | HEX | JL generated |
| FL1251 | S0614 | GCAAACGAATACTGGCTGTAAAGA | TGTCCACCTGATGGTTGTCTGA | HEX | JL generated |
| FL1262 | S0108 | TTTATTTGGAATTAGTGGGGTTA | CATTATCGCTCACATCGCTCCT | TAMRA | JL generated |
| FL1267 | S2721 | CATTGGCGGTTCTGCGG | GGAAATGGTAGTGCAAGGGGA | TAMRA | JL generated |
| FL1269 | S2529 | GGGTGAAAAGTGATGACCAAAAC | CCAAGGAAAATCCCTGGAATCT | TAMRA | JL generated |
| FL1277 | S0821 | CGTTTTGTTCCGCACGATTT | GGTAGTAGGAATGTCAAGATTACACGTT | TAMRA | JL generated |
| FL1278 | S1317 | CAGTCGTTGTGGTGAGGTTTGG | GCCGTATGCTCTTTTGTCTTCC | TAMRA | JL generated |
| FL1281 | S2218 | GTACTGTAATGATTGTAATTTGTTTTGTGC | TCTCGCCAACCAATCGCTT | TAMRA | JL generated |
| FL1301 | S0326 | GGGCTGCCACTTACATCATTTAG | GTAGCCCATGTAAATCTAGCTCAAATA | FAM | NF generated |
| FL1302 | S1716 | GAAATACAAATGGCGGGCAAT | CAACCTACGGATAATAAGGAATGACA | FAM | NF generated |
| FL1303 | S2025 | CACACAATGGACTGGCTCTCTCTTA | AGAAACCGATTGCCATACTGAAA | FAM | NF generated |
| FL1305 | S2445 | TCAATGTATGCAATGTAAGCGTGT | CGTCTGCTCCGTCTCCACC | FAM | NF generated |
| FL1307 | S1024 | TGCCGATGCTAATGCGATAAT | GATCACAGACTAAATGTCACCCACC | FAM | NF generated |
| FL1308 | S1150 | TTGACTCTGCGGGTTTAACATTT | GGGGTCGCCACAGCTAATTT | FAM | NF generated |
| FL1310 | S0324 | CGATGGAGACAGTTGGTTGACGA | ACTTCGATTCCGCATGTTACAA | FAM | NF generated |
| FL1311 | S1821 | GCACGCACGCATTAAAACAC | TCGGACTGCGGAGTAGTTGGT | FAM | NF generated |
| FL1312 | S1918 | GATTTTATCAAGCACTAGCGTGTATG | CGGAATAGATAAATAAGGTTGGAAAGG | FAM | NF generated |
| FL1314 | S1917 | CATACGCAGAGAATGACAGACGC | CTTAAACACGTAGATTATTTGTGGATTC | HEX | NF generated |
| FL1318 | S2444 | AGATTTCGCCAAGACTTCGCTA | CGCGGAAGGAGGAGTATGAA | HEX | NF generated |
| FL1319 | S1222 | ATAATAATGGTTCAGTCCTCATAGCAA | CAGGTAGTCTTATGAGGAGTCATGGA | HEX | NF generated |
| FL1320 | SX03 | CACAACAATAAGGATACAGAGTACAAGG | CAAAACCATAGCAGCGTTCAAG | HEX | NF generated |
| FL1321 | S1516 | TGAGTTGTCGTCAAAGCAAAATAAA | TTGCTTCGTCTCGTTTCATAGGT | HEX | NF generated |
| FL1324 | S2434 | TCTTCTTTAAGCGTCCACTCCATAT | TGCGTGGACGGAGGAGTATG | HEX | NF generated |
| FL1325 | S1324 | TGATGCACAAAACAAATTACAACCA | GTAGATTGGGTGAGATTGTTGCC | TAMRA | NF generated |
| FL1326 | S0221 | GACAGAGAGAGCGACTTTGTTTTATG | CCTGGGACAACAAGTAGCCGT | TAMRA | NF generated |
| FL1328 | S1428 | TTTGAAGTCAGATGATTGGGTGTC | GAAATGTTTGGCGATTACCTACG | TAMRA | NF generated |
| FL1329 | S2329 | TCGCGTTCCCGCCAAA | TCGTTTCGTGCGTGCCAG | TAMRA | NF generated |
| FL1331 | S0615 | CAAGAGTACATTAAAGGGCAGTGC | TGTAGTTGTGACGTAGGGGCTG | TAMRA | NF generated |
| FL1332 | S2433 | TCTCAAGGTGGGTGGCG | ACTAGACATCCGATAAACGAAGCA | TAMRA | NF generated |
| FL1334 | SX05 | TGACCGTGACGCCCGC | TGCGTCACAAAACTAATTATAGAAATTT | TAMRA | NF generated |
| FL1338 | S1144 | TCAAGGTGGGTGGAGCATTT | CGCCTGTTGTACCTATTACTATGGG | FAM | NF generated |
| FL1339 | S1325 | CCAGGCTCCCTCCTTCTT | TGCCAAAAGTCGTTGCTTATAATC | FAM | NF generated |
| FL1340 | S2223 | GCGACGCACTCCACCAAG | TATGTATTTCACGGTAAGCAGCG | FAM | NF generated |
| FL1344 | S1824 | CATTTGATGTTGGGATAATTGCC | TTAGCGATAAGACCGCCTATTGTA | FAM | NF generated |
| FL1345 | S1148 | CGCTCTTCGTCATAAATAAATCTG | ACTTCCACCCGCTATCCACA | FAM | NF generated |
| FL1346 | S1025 | AACAAAAGTCCTATACGAAGGCAAC | CGTCAGACCTGGACAAAGAAAGT | FAM | NF generated |
| FL1347 | S1617 | TGTAATCGGTTCACATCGCTAGA | TTTGGCAGCTAAATATCTTGATAATGT | FAM | NF generated |
| FL1348 | S1023 | GCGAGTGGTTTGTTCGGATTC | TCGTGACTCCGCTTGATTCC | FAM | NF generated |
| FL1349 | S1326 | CGTGTAAACGACTAAAGATACGGGTG | CCTTAATCACAAAGAATGCGTCC | HEX | NF generated |
| FL1350 | S1321 | CTAAACACTGTCAATCTTACAAAGCAAT | CCACGTTTTCAATAACAACACCAA | HEX | NF generated |
| FL1352 | S1513 | CATAGATTTTCATACACAACCGTTTTT | ACTGAGTCAAATCGTGATAATGTCTGT | HEX | NF generated |
| FL1353 | S1147 | CCAGATTTCTATGGGCATGAGTTA | TGAAGGGGCGATGTACGAAA | HEX | NF generated |
| FL1356 | S2533 | GGCTCTTAAACTTAGACTATTTTGAAACA | CCAGGTAAAGAAAGTTAACCAGGAAG | HEX | NF generated |
| FL1357 | S1429 | AATATTCCCTGACTTTTGTGCTAG | CCGCTCGGGACTATTTTGTCTT | HEX | NF generated |
| FL1358 | S2630 | CACCAGGCTACAAAACGGGA | CCCCGTGAGAATGAGATGTTTT | HEX | NF generated |
| FL1363 | S1430 | GCTTCCCATAACATAGGGCAACT | CCTACTAGGCAGCGGTGGATT | TAMRA | NF generated |
| FL1364 | S1713 | ATTCAAGTGCAAGTAAACTCGGC | CGAAGGAACGAAGCAAAGAGGA | TAMRA | NF generated |
| FL1365 | S1514 | AGGCAAGGTGGAGGGACG | CGAAACTAACCCAAAGATGGACAG | TAMRA | NF generated |
| FL1367 | S1919 | AAAATATCGAATACGGAACAGTTAG | GTTCTTGGCGGTGTATGTGCT | TAMRA | NF generated |
| FL1371 | S1616 | GGTCAGACGCAAAGTGCCAAG | AAACGCCGCCCGACATT | TAMRA | NF generated |
| FL1401 | S2120 | TAGCGATAAGACCGCCTATTGTAC | CAACCAAAGCAAACAATGTAAAAGT | FAM | NF generated |
| FL1403 | S0824 | AATACCCGCTTCGTCAAAACAC | CGAACACGACCGACCGAGAT | FAM | NF generated |
| FL1405 | S2629 | AATTACAAAGCGATAAACGACAGC | CCTGGCACTTCTAATCCTGTTTC | FAM | NF generated |
| FL1406 | S1432 | TGAAGAAACGAAGCACTGAGTTGT | GGCGTGTTGTCGTGGTCAGA | FAM | NF generated |
| FL1407 | S0918 | CGGACCTAATAGCGGAGGAACT | TTAGCGAGATTAACGAACAGCAA | FAM | NF generated |
| FL1408 | S2330 | TGTAGAAAGAAGCACGTAGTCCGA | GCGAACGAGATGACAACAGGAT | FAM | NF generated |
| FL1413 | S0825 | GTCATTCAGCAAAGCGGCA | CGTGCATGATTATCCCAGCG | HEX | NF generated |
| FL1415 | S2627 | GACCAAAGAATCGAAAGCCCT | CAGTTTTACCATAACCCACCCAC | HEX | NF generated |
| FL1418 | S1621 | GAACTCGTCCACCCATCTAAGC | GGCCATTGTTCTACTTGCTCTGC | HEX | NF generated |
| FL1421 | S0616 | CCGCTGTTTTGAAATCGTCCTA | AAAGTTGCTCGCTCATTACCTGT | HEX | NF generated |
| FL1425 | S2224 | CTTGCGACAGTAAGTGCTAATGGTA | GCAAATTATTACACTACAGAACTTCTGTGA | TAMRA | NF generated |
| FL1428 | S1322 | GCAATTCGCTCTACGCATCACA | CAGGTGGACCATATGCTTACGA | TAMRA | NF generated |
| FL1432 | S1620 | CGTCCTCTTACCCTCGTACCTATT | GGAAAATGAAAAGTCCAAAATCAAAT | TAMRA | NF generated |
| FL1436 | SX04 | TGGAGAACAAATTAGCGGGG | AACAAAGTCGTGCTCCGATTACA | TAMRA | NF generated |
| FL1439 | S2818 | CGTTTTAGGAGGTTCAATACAGCG | TCCGACAATGTGATGAATAAATGAA | FAM | NF generated |
| FL1440 | S2534 | TTGTGAATTGATTCGCACTGGA | TTCACCTTTTACGATACCAACTGC | FAM | NF generated |
| FL1442 | S2628 | TGCCGATAGCGGGTAGCG | GAGATCGGTTTTAGCGATAAGACC | FAM | NF generated |
| FL1445 | S2026 | AAGGTTGCGACGGGGAAA | TGAATTAAATTAAGGACAATACAGGCT | FAM | NF generated |
| FL1446 | S0617 | CGCTGCCTGCCCAAGATA | TCCAAATGATTCTCGTCCACCT | FAM | NF generated |
| FL1449 | S1431 | CGGCATTAAACCAATACGGG | GCATTGGTTGTATGGCAAAGTTAA | HEX | NF generated |
| FL1451 | S1920 | TCCCGGCCTTCGACCTAA | AATAAAATCAAACCGGGAAGAAAA | HEX | NF generated |
| FL1458 | S0321 | CGTCAGGTTTGAGCCCCG | TCTTGTCCACCTGATGGTTGTCT | HEX | NF generated |
| FL1464 | S2335 | CGGTAACCATTTAACACCAGATTG | CGGAGTCGCCATTGTTGC | TAMRA | NF generated |
| FL1467 | S0320 | TTTAATGGACAACCAAATTGGACTAC | TGATATGTATTCCCAGTTTAGCGGT | TAMRA | NF generated |
| FL1469 | S1224 | CGGACCAACATCAGACCAAAATAG | CCTGCATTTATGTGATACAAAGGAA | TAMRA | NF generated |
| FL1501 | S0522 | CGACGATGAAGGAATAACATTGAGT | GTTACGGGAGTCCATAGACATCAAT | FAM | NF generated |
| FL1502 | S2119 | TCGAAAATAACTCGTCTGACCGT | TTGATACATTACGTACGGTGGTGAC | FAM | NF generated |
| FL1503 | S1223 | GTATTTGTGCTACGGGTAACGAGA | CATTGCTCATTTTGTTGGTGTTCA | FAM | NF generated |
| FL1505 | S1618 | AGTCAGTGGTTCAGTGCTAGGTCA | AGTGGACGAGCTCAACATACGAT | FAM | NF generated |
| FL1506 | S1323 | CGCCGATTGATTGCTTACAGA | GCCGTCATCTCAATACTCACTTCTC | FAM | NF generated |
| FL1508 | S0826 | GCGATAAGACCGCCTATTGAACA | TCAGCAAAGCGGCAGAGC | FAM | NF generated |
| FL1509 | S0523 | TGAAATGCAGGCGTATGAGAAAT | GCTGGTGAATGCTTGACGAGTG | FAM | NF generated |
| FL1510 | S0222 | TGACCCATGTACTCATTTGTAATTCTT | GAATAATGCCCTTCCAATCCC | FAM | NF generated |
| FL1512 | S2443 | ACAGCGTGCCCGACCC | GATCGTTTACAGGGAGGGCG | FAM | NF generated |
| FL1515 | SX02 | GCCCCACTGCCAGCACTCT | GCTTCCCATGATTAAGGAGTTGC | FAM | NF generated |
| FL1516 | S1619 | GCACGTACCTCGGCTTAAACAT | ATTTCTTCCAGTAGGCTCGCAG | FAM | NF generated |
| FL1521 | S1517 | CGAATCGGCGAATCCAAGG | TGGACATGTGATGCGTAGAAAGAA | FAM | NF generated |
| FL1526 | S0217 | TTCAAGGTGGGTGGTGGTATTT | GCTATTGTAAACTGTGCGGCG | HEX | NF generated |
| FL1528 | S1827 | TCGACAGACTCGCTTAGCTTCA | AATGCGGTTTATAAGCAACTCTACAG | HEX | NF generated |
| FL1530 | S0223 | TCCTGGCTATAACAACCCCTTCT | AGGTCATTCACAAGTTGTCTCCATT | HEX | NF generated |
| FL1532 | S0216 | CCGCACAATCAAAAGCCG | CGCACGGGACCTATGCTTCT | HEX | NF generated |
| FL1533 | S0919 | CCAAATGATTCTTGTCCACCTGA | TCTATGGGCTCTGTTGACCACTTA | HEX | NF generated |
| FL1535 | S1221 | TGATGAGGGAACGAAATGATAGC | GTCAACCTGATGTTCTGTGGCA | HEX | NF generated |
| FL1536 | S0521 | ATTCGGGCGTAATGCGTTT | TTCACTCATGTAGGTTAGGCATCTG | HEX | NF generated |
| FL1538 | S2333 | GCGAAGCCACTGCCTACCG | CTATTGTAAGCTGTGCGGCGT | HEX | NF generated |
| FL1542 | S2438 | TTAACTGGCTACGAGATTGAGACCT | TTCACTGGATTGGTACACCGC | HEX | NF generated |
| FL1543 | S2331 | TTACATTACATTTTACATTGGGTTCG | GCCTACCGCCATCTAGTTGCT | HEX | NF generated |
| FL1547 | S2219 | TTTATTACACGATCTTATTCCTTCACC | TCATCGCCGTCAGAGTTATCG | HEX | NF generated |
| FL1548 | S1828 | TGAATAAATAGCGAATAATTAAAGAGCAT | CTTTATCTGTCCAGAATTTCGGGT | HEX | NF generated |
| FL1550 | S1152 | AATACTTGTCAGCGACGCACCT | CGCTTCGGAGGTCATCGC | TAMRA | NF generated |
| FL1551 | S1622 | CCCGTCGGACGATTCCTTTT | CCCTGCCTATTACAGCCGTG | TAMRA | NF generated |
| FL1553 | S0916 | AATCTTCTTTTCCTCCACCTTATCC | TTTCAATAATGCTTAACGGAGTGC | TAMRA | NF generated |
| FL1555 | S2222 | CCACTCGACGAATGCAACCA | CGGTCCAGTAGTTTCGGAGCCT | TAMRA | NF generated |
| FL1556 | S1154 | CGGTCTGAAGGGTGGGGC | GCCAGACTGACCGTCGCTTT | TAMRA | NF generated |
| FL1558 | S1823 | CGAGGCGGCAAAGCAAT | TTTTCGTATGGCAGCGAGTTC | TAMRA | NF generated |
| FL1563 | S2531 | GGGGCTGAAGACGCCAATT | CGTAAGCATGTGCAGACACCAAGT | TAMRA | NF generated |
| FL1565 | S1146 | CGTTTCCCCGTTCATTCGT | AATACCACCGCCACCGCT | TAMRA | NF generated |
| FL1566 | S0220 | GTGCGTCAAATACATGGTATGTAGTG | AGTGGCGGTTCGTTTCACATAT | TAMRA | NF generated |
| FL1569 | S0915 | TCAGAATACAGTGGGCAACGAC | ATGGTAGTGCAAGGTAGAGGGG | TAMRA | NF generated |
| FL1570 | S0325 | TAATCACGTCGGACACTTGCTC | GCTGGATTCTCAATTAAATGACGTT | TAMRA | NF generated |
| FL1602 | S0322 | AATGCCGTGTCCAAATTACCTG | CAAGACAGGGTAAAGTGGAGACAAC | FAM | NF generated |
| FL1603 | S0914 | TGAAAGTTAGAATGGAGGTTAGGAAG | TCTTCTATCAGCCGTCATCTTATCAC | FAM | NF generated |
| FL1606 | S0218 | CCAAATGATTCTTGTCCACCTGAT | GATCAGCCGAGGTGGAATTACTT | FAM | NF generated |
| FL1608 | S0219 | CCGCCCTACGCCGACTG | TGTAAATGAAATGGTTGTCTGAAGG | FAM | NF generated |
| FL1609 | S2225 | GCCTGCGACACGAAACTGA | GGCGACGGCGACCTAAA | FAM | NF generated |
| FL1610 | S1149 | TTTGGTTCGCTTTTATCTTTAGTGA | GATGCGTTCCGCGTCGT | FAM | NF generated |
| FL1612 | S2446 | ACCGGGCAAATCTGGATCA | GCCAGCAGCTTCATGTCCTC | FAM | NF generated |
| FL1614 | S2221 | GCTCGTCAGCTTCGTCTACATG | GGCACCGTGAACACCTCCG | JOE | NF generated |
| FL1615 | S1518 | GGATAGCTGTAATGTACGAGCGAAA | GGCACTTCAGGTTTCTGTCGC | JOE | NF generated |
| FL1616 | S1712 | GGACAGATGGGCGAGCAAT | CTCGCCCACCTCTTCTTCTTT | JOE | NF generated |
| FL1618 | S1715 | CAGCAAATACAGCGAGGAGC | TCGTTGTAGGTAGCATGTATGTACTATTTA | JOE | NF generated |
| FL1621 | SX01 | GGGTGGGTCAGCGACATACG | TTGGTTCATTTTGTTGATTCGCT | JOE | NF generated |
| FL1626 | S2332 | TTGCATGTCGTACAGGCTTCAG | GTGGCTGGTGTTACTTTGTTATTTG | TAMRA | NF generated |
| FL1628 | S2530 | GGTCGCACGGTCGCCTA | AGGTCATTAGGTTATCATTTAAGGTCAT | TAMRA | NF generated |
| FL1633 | S1825 | CCCCTTCGCCCTAAACGC | TTGTCTATGGTACACCGCAACATAC | TAMRA | NF generated |
| FL1635 | S0823 | AACATCGGCACAAGTGTATCAAGA | GCTGGCGAGATGAGAATGGC | TAMRA | NF generated |
| FL1636 | S2437 | CGAACAGATTGCGATACAGTTATTTTA | AGTTAGAGGTTGCTTTTGAATATTTACAT | TAMRA | NF generated |
| FL1639^1^ | S2442 | AAGACAGAGCGAAGTGGAGGAG | ATATGAAGAGGAGCAAGACAAAGAAA | - | NF generated |
| FL1640^1^ | S2436 | GAAATTACCATCAATGGAGGACG | AGCATATCGCTACGGTAGAATCG | - | NF generated |
| FL1641^1^ | S1714 | GGCATTAAGCCCGCCAAT | GGAATGAACTACTTCTTTGTGATTGATA | - | NF generated |
| FL1642^1^ | S2220 | TCAGTGTAATCTTTAGAAAATCATCGC | GCGGAAAGAGCGGACGGT | - | NF generated |
| FL1643^1^ | S0917 | CGGTCCGCTAGGATGACTCAAT | CAGGCATGAAGTGTTAGGTTTAGGT | - | NF generated |
| Markers for gene mapping | | | | | |
| Rp0201 | Rp0201 | ACCGAGATCCTTGTCCGTAT | CAGACCCCAGGTGATTGAGA | - | Gene-based |
| Rp2001 | Rp2001 | TGCGGGTTTGCGTATTC | TTGCCAGGTTTGTTTTGTTAT | - | Gene-based |
| Rp2501 | Rp2501 | GGAACCCGATGTATTAGATTTG | ATTACTATTGAGATTGCGACCTT | - | Gene-based |
| Rp2602 | Rp2602 | CTCTTAGGATAAACGGACGAAT | TCTTACAAGCACATCAGCACC | - | Gene-based |
| Rp2701 | Rp2701 | ATCAACTTGTTCACCACCTCC | TCTCCTAAACCTCCCCTTCA | - | Gene-based |
| Rp2902 | Rp2902 | TTTCCAGTCATTACCTTCACATT | CATTCCATATCATCTCATCTCATT | - | Gene-based |
| Rp3401 | Rp3401 | GACCTTTCAGGACAATACATACAA | CACGGCGACTGCGACT | - | Gene-based |
| Rp402 | Rp402 | GAGGGCTATTCGTGCTGTT | TGCTCGTATCCGATTGGTC | - | Gene-based |
| Rp5401 | Rp5401 | GTGATTTGACCGTGCTGATTTT | CTTTGTCTTTGTACCCATAGTTCCT | - | Gene-based |
| Rp5701 | Rp5701 | AAGCGACGGAATGGAATACA | CGTGGCTAATGGCAGATGAT | - | Gene-based |
| Rp6801 | Rp6801 | GCGTTGGACTTGTTGCCG | TGCCAGCGTTATGCCGTA | - | Gene-based |
| C41 | C41 | GACATCACAAGCGTGGACAT | GCTCGTTCAGGCTAGAGGTTA | - | Gene-based |
| dll | dll | AAAGGCTACATAAGCATACA | GATTCAACACTAAGGAGGAC | - | Gene-based |

1 FL1639, FL1640, FL1641, FL1642 and FL1643 were not labeled with fluorescence, whose results were analyzed on agarose gels.
