# Supplementary material for: An integrated genetic linkage map for silkworms with three parental combinations and its application to the mapping of single genes and QTL
Source: BMC Genomics. 2009 Aug 21;10:389. doi: 10.1186/1471-2164-10-389 (PMC2741490; doi:10.1186/1471-2164-10-389)
Supplement: Additional file 14 — Putative epistatic QTL and their interaction effects (100 replicates). We performed multi-marker joint analysis to estimate the association between markers and phenotypes. This table contains the significant interacting pairs with their statistical power value (at least 20%), mean LOD score, and mean interaction effect in 100 replicate tests. [file 1471-2164-10-389-S14.doc]

## Putative epistatic QTLs and their interaction effects (100 replicates)

| Trait | Position1 | Position2 | Power (%) | LOD | Interactive effect |
| --- | --- | --- | --- | --- | --- |
| Cocoon weight (CW) | S0102-FL1241 | S0302 | 28 | 2.61 | 0.0246 |
|  | FL1277 | S2002 | 20 | 2.58 | -0.0305 |
|  | S1312 | S2711 | 20 | 2.79 | -0.0155 |
| Cocoon Shell Ration (CSR) | FL1262 | S2711 | 36 | 4.65 | 0.0140 |
|  | S0106 | S1136 | 49 | 5.26 | 0.0520 |
|  | S0106 | S1312 | 67 | 4.03 | -0.1318 |
|  | S0314 | FL0435 | 36 | 5.26 | 0.0260 |
|  | S0601 | S2002 | 25 | 3.87 | 0.0806 |
|  | FL1251 | FL1139 | 25 | 4.44 | -0.0349 |
|  | S0609 | S1136 | 27 | 4.94 | -0.1683 |
|  | FL0435 | S0817 | 70 | 4.28 | -0.1266 |
|  | FL0435 | FL0705- FL1139 | 39 | 4.90 | -0.1439 |
|  | FL0435 | S2111 | 71 | 4.26 | -0.0671 |
|  | FL0435 | S2404-S2405 | 21 | 3.01 | -0.0144 |
|  | S0804 | S1701-FL0705 | 62 | 3.71 | -0.1036 |
|  | S1612 | S1701 | 54 | 3.96 | -0.0730 |
|  | S1908 | FL1225 | 23 | 4.06 | -0.0883 |
|  | S2621 | S2710 | 49 | 3.93 | 0.3127 |
| Pupa weight (PW) | S0102-FL1241 | S0302 | 29 | 2.63 | 0.0280 |
|  | S1816 | S2621 | 20 | 2.66 | 0.0025 |
