# Supplementary material for: An integrated genetic linkage map for silkworms with three parental combinations and its application to the mapping of single genes and QTL
Source: BMC Genomics. 2009 Aug 21;10:389. doi: 10.1186/1471-2164-10-389 (PMC2741490; doi:10.1186/1471-2164-10-389)
Supplement: Additional file 15 — Information on all confirmed neighboring SSR markers. The marker name, original site, forward primer sequence, reverse primer sequence, and detection results are shown. [file 1471-2164-10-389-S15.doc]

| Marker name | Original site | Forward primer sequence | Reverse primer sequence | Detection result |
| --- | --- | --- | --- | --- |
| NS0101 | S0101 | ATAATCAGCCAAAACGGTCC | CAGAAGCCAGATAATCCAAAGA | Polymorphic |
| NS0101-1 | S0101 | GGGCAAGGTAGAAGGGAA | GCCGAAACAAACAGACAGAC | Polymorphic |
| NS0101-2 | S0101 | GAAACTGAATGAAGACCACGAC | CCGAAACAAACAGACAGACAAA | Null allele |
| NS0101-3 | S0101 | ATTGAGGAGTGTTTTCGGTGT | CGGTTTTAGCGAGTAGCAGA | Confirmed |
| NS0101-4 | S0101 | AAACTCGCACTAAGCACA | CAAGCCGTTACCTACCAT | Null allele |
| NS0104 | S0104 | ACGGTTCAGTTATTTCAGA | TAGGATGTTATTGCGAGTA | Polymorphic |
| NS0105 | S0105 | GCAGTGAAGAGTAGAAAAGGGA | GGAGAAGTAACGACAACCAAAC | Polymorphic |
| NS0106 | S0106 | GCGGACGGAGGAGTATGAA | GGTTTGAATGCTGCTAAGTTGT | Null allele |
| NS0106-1 | S0106 | TTGTTAGTTTAGATTGTCTGTGCCC | CACCTCGTCACGCTTATCC | Confirmed |
| NS0201 | S0201 | CTGCCTACAAGGGTAATAAA | GTCGGGAAGAGTCATAAAGT | Polymorphic |
| NS0206 | S0206 | ACCTCTGGTTGTTGACTGTTTT | CGTAGTATTCGTGGGTCGTAG | Polymorphic |
| NS0206-1 | S0206 | GATAGGATTGCCTGTTGTC | TCGTAGTATTCGTGGGTC | Null allele |
| NS0206-2 | S0206 | GTATCTTCGGAACCTTGC | TGGGTCGTAGTTACCATTAG | Null allele |
| NS0207 | S0207 | CTGAATCTTCGCACCTTACAA | CAACGACTGCCTTACCCTT | Null allele |
| NS0207-1 | S0207 | GTCTGAATCTTCGCACCTT | CAACGACTGCCTTACCCT | Polymorphic |
| NS0207-2 | S0207 | CCTGCCTGTTGACTTATTT | TGAGTTCTTACCGCCTTT | Polymorphic |
| NS0208 | S0208 | AAGAGGACGACCGAAGAA | GATGCGGGAGAAGTGAAT | Null allele |
| NS0211 | S0211 | TGGGCAAGAGTGGTGAAGG | GGTTTGGGTCCTTGATTGGTA | Polymorphic |
| NS0213 | S0213 | ATGGTGGAAATATCGGCAA | ATAGAAAGGGCGGCAGACT | Polymorphic |
| NS0214 | S0214 | AGCGGTTAGTTGCCCTCA | CGTTGTCGCTTTGACCCTC | Polymorphic |
| NS0214-1 | S0214 | TCCCCACCATTATCTACTGAAC | ATGACTCATGCGGCGTTAC | Polymorphic |
| NS0217 | S0217 | GATGCTTCTTGCTCTTGTGGT | TGGGTCGTAAGTTCGTTTCTG | Polymorphic |
| NS0219 | S0219 | ATAGCCTACCAAAGCATACCAA | CAATCCACCACTACCAAGACAC | Polymorphic |
| NS0219-1 | S0219 | TGAGGGGTTAGAGTGGACGG | TGTAGAAGCCTGGGAGGTGAT | Polymorphic |
| NS0222 | S0222 | TTGGCGACAGATTGTTCATT | TAACTGTTGATGCTGATACCGA | Polymorphic |
| NS0222-1 | S0222 | CCAGCCCAATGCCACTT | CGTCTAACAGCCCGAACAAT | Confirmed |
| NS0223 | S0223 | TGTTGGATGAGCAATAGTAGGC | ATGCGAAGGATGGTCAGATT | Polymorphic |
| NS0301 | S0301 | CGTCTACCTCCGAAAGCAA | CGACAGACCTCAGACACCCT | Polymorphic |
| NS0302 | S0302 | GCAGTGACAGTGCTCCAA | AATGCCATCTGACCAACA | Polymorphic |
| NS0303 | S0303 | TACGCAGAACAACTACCG | GCTGGCTACAACTACACG | Polymorphic |
| NS0306 | S0306 | TTCTATTTCATTCGCCTCTATCAGC | GGCATTCTTTCCAGCCACAT | Polymorphic |
| NS0306-1 | S0306 | TCGTCATTCTCACATTTCATCC | CTCGGTCTAAACTGTTGTATTCCTA | Null allele |
| NS0307 | S0307 | TTCCGCAATCGTTAGCAT | GCCACAATCCCTTTACCC | Confirmed |
| NS0307-1 | S0307 | CAGGTGGTTATCGGAGAC | TTAGGCTTGCTGTTATGC | Confirmed |
| NS0309 | S0309 | CTATTCCTTTGTCTCATTCGCA | GAGTAAATCAAGCACGGTCATC | Polymorphic |
| NS0309-1 | S0309 | GCTTCGTAGAGCGTCATA | AAATCCTAAAGTTGCCTTC | Confirmed |
| NS0310 | S0310 | ACTATTCACTTGCCCTCGTTC | ACTTGTTCTCGTCTCGTATCTGT | Polymorphic |
| NS0311 | S0311 | TGCGTAGATAAACACCACCAC | CTTCATTGATAGCATTAGCGTC | Polymorphic |
| NS0312 | S0312 | GAACGGTAGAAATAGGTAGGCG | GGTGAGTGGCGAAAAGACAA | Polymorphic |
| NS0314 | S0314 | ATACAACGCCTCCAAC | ACCAACCGAAACCTTA | Polymorphic |
| NS0315 | S0315 | GAAGTTTGTGCTGGAAAAGTTG | GGTTGGTTGGTTGTTTGGTAG | Null allele |
| NS0315-1 | S0315 | GACGAAGAACCCTAAAACTGC | CAAACGAGGCTTGTGGAGA | Null allele |
| NS0316 | S0316 | AATCACGAAGTCTGGATCAAAG | CACTGTACTGCTGGTAATGCTAA | Polymorphic |
| NS0317 | S0317 | CGCTATCATACTTCACAAGATTACC | CCCACCATAGACGACCAAG | Polymorphic |
| NS0318 | S0318 | TACATAGCCCACCCGACAC | GGCAACATTGGAGGAAGC | Polymorphic |
| NS0323 | S0323 | ACCACAGACGACAAGAACAAAA | CAGGTCACCAGGTCCAAGAG | Polymorphic |
| NS0324 | S0324 | CCAATAGATGTCGGGAAGAGTT | GAATAGCCCCTTAGGTTAGCAG | Polymorphic |
| NS0325 | S0325 | ATGTTTTGAGGTCGGTGGC | TCGTTGGATGCGGAAGG | Polymorphic |
| NS0326 | S0326 | CTTGAGACATCAGAAGCACGA | CCAGGAACTACAGCCGAAA | Confirmed |
| NS0401 | S0401 | CGCTTCAAGCTGTAGCTGTATT | CCTATGTCCTTCTCCTGGTTCT | Null allele |
| NS0401-1 | S0401 | CGCTTCAAGCTGTAGCTGTATT | AGTATCCTATGTCCTTCTCCTGG | Null allele |
| NS0401-2 | S0401 | CACGCAGTCAGTAGTAATCG | CGGTATGGGTAGCACAAT | Confirmed |
| NS0402 | S0402 | TCGCCTTACGAAACTCCTG | CGCTTCTGGTCATACAATCAA | Polymorphic |
| NS0403 | S0403 | TGTTACCTACGAGTGGCTACGA | TTGCCTGCTCAGTTTCCTTC | Polymorphic |
| NS0406 | S0406 | AAGCCAGTCGGATTGTTTG | TGATGGTAGGTGATGACGGA | Polymorphic |
| NS0407 | S0407 | GGATAAAGACGCCGTGATT | GATTGCTATGACTGGCTGTTG | Polymorphic |
| NS0407-1 | S0407 | AAAAGGTCGTGGTAAAAGGC | TGCTGAGTGGCTAAAGATGTG | Confirmed |
| NS0408 | S0408 | CCTGGTGTTGAGGCGGTTA | ACAGTGCGGTCTCGTCTTGC | Polymorphic |
| NS0408-1 | S0408 | CAAGTGATGGAGTTATGTCTATGG | GCCGAGTTTCGCAGTTTT | Null allele |
| NS0409 | S0409 | GTTTCTTGCTCGTTGG | GACTTCGTAGTGCCTCA | Polymorphic |
| NS0409-1 | S0409 | AGAGGTCCCGCAGTAG | GACGGAGGAGTATGAAA | Confirmed |
| NS0410/11 | NS0410/11 | CGCATTAGTTGGACGCAGA | TTAAGGATCACCAGTGAGAAAGAC | Null allele |
| NS0410/11-1 | NS0410/11 | GGGCGACAATCATAAC | GGACTGACCTCGTAAAG | Polymorphic |
| NS0410/11-2 | NS0410/11 | TTAGACAACAAAGATGCCCC | ATTCAAGCGACATTCACCC | Null allele |
| NS0413/14 | NS0413/14 | AACATCTGGTGGGTCAACATT | TTAGCCGACTAGCGTCTTACA | Confirmed |
| NS0413/14-1 | NS0413/14 | TCTGGTGGGTCAACATTTACG | CGGAGTGAGAACGAAGGCAT | Null allele |
| NS0416 | S0416 | TAACCTAAGCCAAGATTCACCA | GGGATAAAACAACCAACCGC | Polymorphic |
| NS0418 | S0418 | CCAAATGATTCTTGTCCACCT | CCAACTATCTCCAACTGTGACG | Polymorphic |
| NS0419 | S0419 | TATCGTTCCGACGCTGACT | CCATACGCTTTTACGCCAT | Polymorphic |
| NS0502 | S0502 | CTGTCCTCATCCACTTATTGCC | TGTATCCAGGGTTGCTTTCG | Null allele |
| NS0502-1 | S0502 | TCTACCCTGTCCCATCTCGT | TGTCGTCTGCGTCTGAAAAT | Confirmed |
| NS0502-2 | S0502 | TACGGTTGTTTACGATTTGGTC | CCGAATGTGCTTTCCTGC | Confirmed |
| NS0505 | S0505 | GGGGTGTTAGGTTTTATTTTCG | AAGTGGCACAATCGGAGC | Null allele |
| NS0512 | S0512 | GATTCAAGCCCTACACCCC | ACCCTGCTGCCATAAAAGAT | Confirmed |
| NS0514 | S0514 | CGTGCTTGATGTTTGTGGG | CTCCTCTTTGTGCGACTCTTC | Confirmed |
| NS0515 | S0515 | GCAGTGTTTGAAGTCTATTGTTACC | CCCACATTGCCACATTGA | Null allele |
| NS0516 | S0516 | TGATGGAGTTTAGTCGGGG | TGATTGAAAGTTTACTGGTGGC | Null allele |
| NS0517 | S0517 | TTCTTCTGTTTCGTTGGTGTTC | GATCTGGCGATTGCGTTTA | Polymorphic |
| NS0519 | S0519 | GTGGACTACGCCGTTTCAA | TTAGAGGGGTGCCAGAGGT | Polymorphic |
| NS0520 | S0520 | AGAGGGATGAGGGGAGGAA | CACAAGAGGAGGTGGTAGGAAC | Polymorphic |
| NS0521 | S0521 | ACGTAATGCGAGAACATCAAAG | AGGAAGAACGGTCCGAAGA | Confirmed |
| NS0522 | S0522 | CAGGACAAAGACGGAAAGAAG | AACTAAAGTGAAGGCAATGGTG | Polymorphic |
| NS0523 | S0523 | TGAAAATGCTGAGGTGGATG | AACCGTGGTGGATTATGAGTG | Null allele |
| NS0602 | S0602 | GCTTCCTTGGCAGTTTCC | GTACCCTTTGCGACTTCAGA | Polymorphic |
| NS0603 | S0603 | CCCATACTCGTCTTGTTTGTCA | TGAAGCGTTTAAGACGAAGGT | Null allele |
| NS0605 | S0605 | TGATTTGCCACCACCATTG | GGATCTTAGAGTGCTCCCCAG | Null allele |
| NS0608 | S0608 | CCGAGCGAGTTACAGCAT | AACGCCTCAACGCCTATT | Polymorphic |
| NS0608-1 | S0608 | CACGGTAATGATAGGTTGA | GTGATTCTAATGCGAGGA | Confirmed |
| NS0612 | S0612 | TGCCTAAATAAAGCAACAGA | TACCTACCTACACTAACATACCG | Confirmed |
| NS0615 | S0615 | ATGTAGTGGTGGTAGTGGCGT | TTGGTAGTTGATTCCTCGTGAT | Confirmed |
| NS0701 | S0701 | CCTACCCTCATTTATACCC | CGATTCCTACATATCCCAC | Confirmed |
| NS0703 | S0703 | CTCCGCTCTTGTCGTGCTT | CTGACTTCTTGTAAACCATTCTTCC | Null allele |
| NS0704 | S0704 | ATTCCGCACTCCTTCTC | TCACCGTTCTCGTTGTT | Polymorphic |
| NS0705 | S0705 | CGATGGTATTCTATTGCTTGGC | TGACGTTCACGCAGCTTTC | Polymorphic |
| NS0706 | S0706 | AATCGTAACACCATTGCCAT | GACAGCGTTGATTGACTTCG | Null allele |
| NS0708 | S0708 | CATCATTCTCCTGCCCTTCT | AACTCGTTAGATGTAAGCACCTG | Null allele |
| NS0709 | S0709 | GTGCCCAAACTCTGCGTAA | TGGCTTGCTTTCTATGAGTGAG | Null allele |
| NS0710 | S0710 | TAGCCGCTTAGGAAGAGTTTAG | CCATCACCAGAATCGAAGTTTA | Polymorphic |
| NS0711 | S0711 | TAGTCGTTGGTGGTTAGG | CAGTGACCGTTGCTTGTA | Polymorphic |
| NS0801 | S0801 | TGCGTGATGTAAAGAAG | GGACCGAAATGATGAA | Null allele |
| NS0802 | S0802 | GAAAATGGTAGTGTAATGTAGAGGG | AAGCGACCGTGAAAGGAA | Polymorphic |
| NS0806 | S0806 | CGGTAATACTTTTAGTGCGTCTG | GCAACTCATCACCTGTCCCT | Polymorphic |
| NS0808 | S0808 | GTAGTGTAGAACGCCCTCCTG | TGTGACCTCGTCCACCCAT | Polymorphic |
| NS0808-1 | S0808 | GCAACCCTCCTTTACTCCAC | TGACAGGCTAACAACCCACA | Confirmed |
| NS0810 | S0810 | TTGATCCACCTCCTTAACCAT | GAACACCGATACTCGCAGC | Polymorphic |
| NS0811 | S0811 | GACGTTCTCGAGTTTTAGCG | AGGTAGACTGGTAGGTAGATGATTC | Polymorphic |
| NS0812 | S0812 | TACCTACGAACCACGAAAACC | CACCAGTAAGAAGTACCTACCCAC | Null allele |
| NS0818 | S0818 | GCTCACCTGATGTTCCTTGG | CTAAAACTAACCTGTATCCTTCCGA | Polymorphic |
| NS0822 | S0822 | CGCATACCAAAGCAACGA | ACGAGGCATAGGAATCACAGT | Polymorphic |
| NS0823 | S0823 | CGAGCCCCTAAAACATTCC | TGCCAACAGCAGTCTCCAA | Polymorphic |
| NS0824 | S0824 | GGTCGTCATTCGGTATCATTG | TTCTTGGAGGGGCGTTG | Polymorphic |
| NS0824-1 | S0824 | GGCTGAAGTATCTGTCGGAGTC | CACCTTGGGTAACATTATTGGA | Polymorphic |
| NS0825/26 | NS0825/26 | ACTTCTAATGCGACTTCCAACA | CGACACCTTCTCCGTGCTAA | Polymorphic |
| NS0825/26-1 | NS0825/26 | TTCTGACCGTGGGAATAAAAG | CTCCTCTGGATCGAAATAAACA | Confirmed |
| NS0901 | S0901 | ACTCCCTACACCAGTTGCGG | CGGACAGCCTTTGAAGATGA | Polymorphic |
| NS0901-1 | S0901 | GCGAGGTACACGAATCAAAA | CCAATAGATGTCGGGAAGAGTT | Polymorphic |
| NS0903 | S0903 | GTTGGCACTTCAGGGGAGA | ACGCAAGCCTTTTGGACA | Confirmed |
| NS0906 | S0906 | CGTGGCGTGATTGTTAGT | ATGAGTTCGTGCTGGTGA | Polymorphic |
| NS0906-1 | S0906 | GCTCATCTTTATCCCACTT | GGTTCCACCGAATCTTAT | Confirmed |
| NS0908 | S0908 | TTGGATCAGGAACCGTACAT | CACCCAGACAAAGAGCAAATAA | Null allele |
| NS0909 | S0909 | TAGCATTAGGAAGCAGAGGACA | GAGCACAGGTGGAATAGCG | Polymorphic |
| NS0910 | S0910 | TACCAACACCGATGAGTTAGAA | TTGATTTGAGGAGTAGGGCA | Polymorphic |
| NS0911 | S0911 | CCACCCCAGTGAAGCAGTA | CGAAAATCGCAACAAGAAGA | Null allele |
| NS0911-1 | S0911 | AGCGGAGCAGTAATAAAGAATG | TGACAAATACACGCACAGAAGA | Polymorphic |
| NS0911-2 | S0911 | AGTGAAAGTCTCATCAAAATCGGTC | TAGGGGTTCCACGGTAGTCG | Polymorphic |
| NS0912 | S0912 | ACTACTGCTACTACTGCTGTTCCA | TGATTGTGAAGGGGACAGTTT | Polymorphic |
| NS0913 | S0913 | AAAAGGTCGTGGTAAAAGGC | TGCTGAGTGGCTAAAGATGTG | Confirmed |
| NS0914 | S0914 | CGCACGCATAGGCATCA | CGTTCTAAACAGACCTTCCACA | Null allele |
| NS0915 | S0915 | TGGACCGATTTTGACGAGA | GACCGAAGAAGACTGGATGG | Polymorphic |
| NS0919 | S0919 | CACCTGCCCTGGTAAAACTG | CCTGTACTATTCCGAATGCCTAT | Null allele |
| NS1001 | S1001 | GGAAGTCAACCGTGAACATTT | GTCGCTTTCGCAAGTAACC | Polymorphic |
| NS1001-1 | S1001 | AGTGAGTTAGAGCGTTGG | CTATTTCTCGTATTGTTTCG | Polymorphic |
| NS1001-2 | S1001 | CGTTCACCTGTCTACCCAATA | TCAAAGCCACATCGTTCC | Confirmed |
| NS1001-3 | S1001 | CTGAAACGGAGTCACGAT | AAACACGCAAGCACATTA | Polymorphic |
| NS1003 | S1003 | ATCCAACCACTCAGGCGA | CTGAACCAAGCATCCGATT | Polymorphic |
| NS1005 | S1005 | GTCTACAATCCTTTATGCGTCA | CGAGCCTCAAATGTTCCAA | Null allele |
| NS1006 | S1006 | CGTTTTACTGGTCTCGGTCTT | GCCCAAATATCAACTCTGCTAG | Polymorphic |
| NS1007/08 | NS1007/08 | GCTCACAATACGCCCTACAA | CACCGTTCATAACACGCAAA | Polymorphic |
| NS1007/08-1 | NS1007/08 | GACATTGTGATGATTCTGGGTT | CTCCGCTATTTTCGTGGTG | Null allele |
| NS1007/08-2 | NS1007/08 | ATTTCGTATCGTCGTTAGTGGT | GTCATAGTGCTCGGAAGTGC | Null allele |
| NS1007/08-3 | NS1007/08 | GTATTCAGAAATCTTGGTGGAGTC | GAGGTAATACTTATGATTCTGGCTG | Null allele |
| NS1009 | S1009 | TGTCGGGAAGAGTTGATTATTG | TGCTGTGGTGAGCTTGAGAA | Polymorphic |
| NS1010 | S1010 | ATTTCATACTCCTCCGTCCTC | CACCCCACGCTTTCTTTAC | Null allele |
| NS1011/24 | NS1011/24 | TTCATCTGGTGTTATGTGGTCA | CGCATTGTTTCTTTTGTTGG | Polymorphic |
| NS1011/24-1 | NS1011/24 | CCTTACCTTGCCTACCTGATG | CCTTGTACTCCGCTTTTGTCT | Polymorphic |
| NS1011/24-2 | NS1011/24 | GGGAAAGTGAAGCGTTTAGTG | GCGGTATTGAGCAAGGGTCT | Polymorphic |
| NS1011/24-3 | NS1011/24 | GATGTATGGAAATGGTAATGGC | GTTGTTTGTGAACTTGTAGTCTGTG | Confirmed |
| NS1011/24-4 | NS1011/24 | ACCGCCACAGGTCATTCA | CACAACAACTTACCGCCGT | Confirmed |
| NS1012 | S1012 | CATCAGACCATTGTCAGATAACG | AGATTACGCCCAGTCACGG | Null allele |
| NS1013 | S1013 | TTCAAAGCAGAAACGAGACG | CTCCACAGGGAAAGCCAT | Polymorphic |
| NS1015 | S1015 | CGTTACCTGGGAATAAATGG | CCTCTGTGGGTCGTACTGTG | Polymorphic |
| NS1017 | S1017 | CGTTCATCGGTAGTGCGTT | GCTCCAGCCAACAGTAACAG | Polymorphic |
| NS1017-1 | S1017 | CAGTCGTCTATCGCAGCAC | TTAGCCAAATCGGTCCAG | Null allele |
| NS1019 | S1019 | ACGAAGGTCCAATACGGG | AGCAGCAGAACAAGCAACTC | Polymorphic |
| NS1021 | S1021 | TGCCTTGGATACGAGTCTTTT | GACACCCCTCTAACTTTTCATTC | Polymorphic |
| NS1023 | S1023 | AGGTTCACGGGTTTTCACG | CCTTTTACCACCGCTCTGC | Polymorphic |
| NS1025 | S1025 | ACCAGTCAACCAAAGGTAGTGC | TATTCGCAACGGACCCC | Polymorphic |
| NS1026 | S1026 | ACCAGTCAACCAAAGGTAGTGC | TATTCGCAACGGACCCC | Polymorphic |
| NS1101 | S1101 | GTTTAGAGCAAGACCCAAAAGTTAG | GCGGTGTCATAGAATGTAGCG | Polymorphic |
| NS1102 | S1102 | GAGAACCGCTCAGTTAGACGA | CTGCTGCGAATCATTTGTG | Null allele |
| NS1104 | S1104 | GTCAACCTTATTGGGTCCTTT | AGAGTAATGCGAACGGGC | Polymorphic |
| NS1108 | S1108 | GAAGTATCACTCACTGGTAGAATCC | GACGCTCATAGAAGACGCTG | Polymorphic |
| NS1110 | S1110 | CGCTCCGATGACTGACGACT | GTTCCTTATGTGGCGATGC | Polymorphic |
| NS1114 | S1114 | AAAGGCGTTCGGTTCGGA | CAATGGTAGCAGTATCTTCAATCAG | Null allele |
| NS1115 | S1115 | AAAGAACCACTCATTGACGACA | GGCATTGCTGACTCCCATAG | Null allele |
| NS1117 | S1117 | GAAATGCCCAGGCTATACGG | GCTTGTGAACATCAGCAACTCC | Null allele |
| NS1123 | S1123 | CAGTAGGTAGGTAGTAGTTACGGTCG | TCTGTGGTCGTCGTGGTCTC | Polymorphic |
| NS1124 | S1124 | GTTGACTTTGGGTTGACTTAGC | CGCCCCGTAGTTGAGATTC | Polymorphic |
| NS1125 | S1125 | GAATCTGAAAATAAGTCGCTGG | CGCTGTCTTCTTCGTCTCCT | Polymorphic |
| NS1126 | S1126 | TGGCACCGTTCTCCTC | TCACCTTTGGCGACCT | Polymorphic |
| NS1127/43 | NS1127/43 | TCTACTGGATGGAGACAGCAAC | GTAACAATGAGGGAGACGACC | Confirmed |
| NS1129 | S1129 | CAGATAGCCAGTCAAGCGAT | GGCAACGGTCAAGTTTCAA | Null allele |
| NS1133 | S1133 | CCCGAAACTGGACTTGAAA | GAGGAAATGAGCAGGATGGT | Polymorphic |
| NS1135/36 | NS1135/36 | GTTTCCTTATCCAACAATACAGACC | GGCGGCGATTCAACTACA | Polymorphic |
| NS1135/36-1 | NS1135/36 | ATCTGAATGGATGAAGCACTGA | CGGCTCGTAGGTTTGTTTG | Null allele |
| NS1137 | S1137 | AGGGAGTAGGAAGTGAGATTTGA | CGACACGGAAGGCGATAA | Polymorphic |
| NS1138 | S1138 | ATAGGCACATAACACCTTCATCC | CGACGACCTCCCCTTCAAT | Polymorphic |
| NS1144 | S1144 | CTCAGTGGGCTTAGTCTATGGG | CCGCAACAATGTCTTCGTC | Polymorphic |
| NS1144-1 | S1144 | TTTCTGTTTGACGATAGATGGC | CTTGCTCTGTTTGACGGTTTTA | Polymorphic |
| NS1145 | S1145 | GCTGGGCAGTTTTCGTGT | GGTGAAGTGGGAGCGTGTAT | Null allele |
| NS1146 | S1146 | TTGAAACGACTTGTCCATAACG | CCCCTGGCATTGCTGAA | Confirmed |
| NS1147 | S1147 | TTCCTTCGCCTACATTCCA | CATCCACAGATTCACTGACACTT | Confirmed |
| NS1148 | S1148 | ATGTCATAAGGCGTGGGC | CTGTCCTGGTGAAACTGAAAAG | Polymorphic |
| NS1148-1 | S1148 | TGCGTTATTTGTCAATGCCC | AGATGTCGTGTCCTGAAGCC | Polymorphic |
| NS1149 | S1149 | GTGAAATCAGCACAGCGAAC | AGGAGATGTATGGGAATGGTAGT | Confirmed |
| NS1151 | S1151 | ATACTGAATGACGAACACGACC | TCAAATGACAATCCCGCC | Null allele |
| NS1153 | S1153 | GACTCTGACATTCCCGTGGT | AAATAGGCAGTGCGGTGGT | Polymorphic |
| NS1154 | S1154 | ACTTAAACGGTTGGCTGGAA | CTCTGTCGTTCTGTCTGTCATTC | Null allele |
| NS1201 | S1201 | TAACGACATACCTCATAAAACGACC | ACCGACAAAGGCGACTACCC | Null allele |
| NS1203 | S1203 | AGTGGTCTAACAACAGAATAAGTCG | TGCGTTCGGTGAAATGGA | Polymorphic |
| NS1203-1 | S1203 | CCTTCACGATTTTCATTGCTTG | GGATGGTAACACCTACTTGGCTC | Polymorphic |
| NS1203-2 | S1203 | CTCTTCTATCAGCCGTCATCTCA | GCCTATGCTCCAGTGTCTAACCT | Polymorphic |
| NS1204 | S1204 | TCAGTTTGGGTGTTCC | GACGGCTGTTTATTGTT | Polymorphic |
| NS1205 | S1205 | TCAATACAAGATTCATCAGCCG | TTCCGTTCCACATCAGTTTT | Null allele |
| NS1206 | S1206 | GGTGGACGAGTTCACAGCCT | TTTTACAACCGACGGGACA | Confirmed |
| NS1212 | S1212 | ACAAGTGGAATGTAGTGAGAAGG | CGGTATCGGAAACCAAGAG | Polymorphic |
| NS1213 | S1213 | AAGCAACTGACAACTTTAGACCC | CCCTTGACAATGTATTACCGAA | Null allele |
| NS1216/17 | NS1216/17 | GGGGTTGAGGATGAGATT | ACTTGTGGAGCATTAGGG | Polymorphic |
| NS1218 | S1218 | GCGATGGCTTCAGATTGTTT | GGTGATGTATGTTGATTAGGAGGA | Null allele |
| NS1218-1 | S1218 | CAGATGTTTGAGGAGTTTGGTAG | ACGAGACGGAAGGTAATGGTA | Polymorphic |
| NS1218-2 | S1218 | GCGTGTATGTGCGTAGTGAGT | GCAAACAACGGTGAATGAAG | Null allele |
| NS1218-3 | S1218 | ATTTAGCCGAATCACCGCC | GCACCACAACGCTGAACCA | Null allele |
| NS1218-4 | S1218 | AGTTGAGATGATTGAATGTCCG | TTGTGAGTTAGCTCTGTATGCG | Null allele |
| NS1219 | S1219 | TGACTGGACTGAGATAACGAGC | AACAACTTTTACACTACCTAGACGC | Confirmed |
| NS1220 | S1220 | AGACAGGGATAGGGATTGTAAGG | CCTCGTATGCGTTGTATGAAGA | Polymorphic |
| NS1220-1 | S1220 | ATACAACCACCCTACTGCCG | GTTATCCTTACAATCCCTATCCCT | Null allele |
| NS1220-2 | S1220 | GTCCCGTGCTAAGTTCGTAAA | CGTGGTCGTCACATCGTCA | Null allele |
| NS1221 | S1221 | ATTGTCTCACCCAACATCCG | CATTGACCATAGGCTTCCATTT | Polymorphic |
| NS1305/15 | NS1305/15 | ATCGCATACCCTCGCCACT | ACAGAGCGAAGTGGAGGAGT | Null allele |
| NS1308 | S1308 | TCGGCACATCGGATTTTA | CTGAAGTTTAAGTGGGCTGG | Polymorphic |
| NS1313 | S1313 | CGGAATGTGAATCGTGTTTT | GCCTTGTGGAGGTTTTGTG | Polymorphic |
| NS1316 | S1316 | GATAACCATTCACCACCTACCG | GGACAAACGAGTTCAGGGACA | Null allele |
| NS1319/26 | NS1319/26 | GATACTCTGTTGCTTTGGTTTCG | GGGCTCAGTGTTGTGGATTG | Confirmed |
| NS1322 | S1322 | AATCTCAGGAACTGCTGGAAA | GTAAGGTCACCGATAAGGGAA | Null allele |
| NS1323 | S1323 | GCTCGTTTATCAGCCTTTTATG | GTGGCAGTTTGTGGTTCAGA | Confirmed |
| NS1324 | S1324 | GACTTCCTCGCAGCATCAA | GCAGACTTCGTAGTGCCTCA | Polymorphic |
| NS1325 | S1325 | TTTCCAGCCACTCTACCTCAC | GCAACCCATTCTGTATCCTGTC | Confirmed |
| NS1325-1 | S1325 | TTGGTGTTAGGAACTGCTATGTC | TCGGAAAAGGAAGGGAATG | Confirmed |
| NS1404 | S1404 | TCGTTTCCAAATGATTCTTGTCCAC | GTTTAGTGGTTCAAGGAAGAGTAGA | Null allele |
| NS1406 | S1406 | GAAATCCTGGGTAAGTAACGC | CGAAGATGTCCAACCTGATAGA | Polymorphic |
| NS1407 | S1407 | CGTAGAGCCTCCAAAGACTG | TCACCTTCAATGCCTTCAAC | Polymorphic |
| NS1407-1 | S1407 | GACTAATACAAGCGGAGCACC | TCCCATCCCACAAAATCAA | Null allele |
| NS1408 | S1408 | CACTGTCATCGCTCCCATC | GTGCTTCCCCGCTTTTGT | Confirmed |
| NS1409 | S1409 | CCTGGGTTCTGACCTTGGA | GGTCGGGTTATTGACATTCCT | Polymorphic |
| NS1409-1 | S1409 | AATCACAAATTCGCCAAAGC | TCAATCGGATGTATGGTTCAGT | Polymorphic |
| NS1409-2 | S1409 | TTGACGGACGACTGGTATCT | TCCACATCGGGCAAACAT | Confirmed |
| NS1410 | S1410 | TGGTGGTGCGGTGATTAG | ATTCCTTTGCGGATTTGAG | Polymorphic |
| NS1412 | S1412 | GTTGGCACTACTTGGTTTTGA | CTCGGAGTTTCTTTGATGGA | Polymorphic |
| NS1414 | S1414 | CTTGATGTGATGATTGAGTAGCG | GCATAGTTAAGTGGGCGTCG | Polymorphic |
| NS1415 | S1415 | CGACATCGTTCACTTTCCG | CGTTCACCAGCCTATCCCT | Polymorphic |
| NS1416 | S1416 | TACCCAACGAATGAAAAGGA | AAAGAGCGGACGGTTACTGT | Polymorphic |
| NS1417 | S1417 | TATCCCGAACCCCAGCAA | GAGGAGAAATGGAGTAAATTAACCC | Polymorphic |
| NS1418/19 | NS1418/19 | GCAACACGCAACTAACTACAACT | GAAGATACATAAGACCACGAACAAG | Polymorphic |
| NS1418/19-1 | NS1418/19 | TTGCTTGTCGCCTGTGAA | CCATCTTGGTTAGTTGCCCT | Confirmed |
| NS1418/19-2 | NS1418/19 | TCCCGCAGTAGTTGAGAATC | CGGACGGAGGAGTATGAAA | Confirmed |
| NS1423 | S1423 | CTTAGAAACAATCTCACGAACTCAC | CAAAACTCAGGCGGGGTA | Polymorphic |
| NS1428 | S1428 | TTTGGATTCGGCGTAAGAG | AAACACTGTGGAGGGGAGC | Polymorphic |
| NS1430 | S1430 | GCACGCTCTAAAACTCTGACC | CAACCAAGGCTGCAATCC | Confirmed |
| NS1432 | S1432 | TATGCCACTGGTGCTTTCC | TCGTCTGATGTTTCGTCCTG | Polymorphic |
| NS1433 | S1433 | GGACATCGGAAGTGAAGGG | TGAGGGGATAGTGAGAACGG | Polymorphic |
| NS1501 | S1501 | GACTCGGGTGATGAAAACAG | GCATGAAACTTCCCACTCTTAT | Null allele |
| NS1502 | S1502 | CTGTGACGGTAGTGGGCTTA | CCTTATTGGGATGGACTTGTT | Polymorphic |
| NS1505 | S1505 | AGAGCGGAAGGTTAGCGTG | CGATGTAGCGGGTTTGATTT | Polymorphic |
| NS1510 | S1510 | CTCTTTTATTGCTACATTGGCG | TCAATAGATGCGGAGGCG | Polymorphic |
| NS1510-1 | S1510 | AACGAAAACGCCAAGATGTA | GGGACCAGGGCAGGATAAT | Confirmed |
| NS1511 | S1511 | CTGTGCCGACCCCACCTATT | GACATCGCATCACTCACTTTG | Polymorphic |
| NS1512 | S1512 | AGATGTCTATGGGCTCCAGTAAC | GATGTGGCGTATAAGCAAAATG | Null allele |
| NS1512-1 | S1512 | CGCCAACCAGTTTTACCC | CTGTCCAGTTCCAAGTATCGTT | Confirmed |
| NS1513 | S1513 | AGAGCAAGTGCCAGCAGAGT | GTATGAGCAGGTGAGTTATTTCG | Polymorphic |
| NS1513-1 | S1513 | CGCTACGCTAATAATGTTTGG | AATGGTGATAAGGTGGTAATGC | Null allele |
| NS1514 | S1514 | GAAGTCGTTTAGTGAGGTAGGGC | CGTAGCAACAATAGCGAAGTCC | Polymorphic |
| NS1514-1 | S1514 | ACTACATCTTGAGAATCGCAGC | TTTGTCCGAACGGGGTG | Confirmed |
| NS1515 | S1515 | GGCAAACACTCCACCCCTT | CCATCCTCCTTTTACGGCTAT | Polymorphic |
| NS1516 | S1516 | GATTACACTCCCGTCTCACCTA | CCAAATGATTCTTGTCCACCT | Confirmed |
| NS1517 | S1517 | ATCTGCTAAAACTGCGGTAAAC | GCACCTTCCGAAACTAAACAC | Confirmed |
| NS1518 | S1518 | TGTCCTCATTTGTAACCATCCA | CTGTAAAGTTCACGCATTGTCTC | Confirmed |
| NS1601 | S1601 | TGTTCGCACTTCACAGACG | GGTGAGCCTATTAGTTCGTGAT | Null allele |
| NS1605 | S1605 | TGAGTTGAGTGAGACTTGAGTTTTC | TTTGGCTATTCCTCTGGCA | Null allele |
| NS1613 | S1613 | GCGAGGATGTTACTGCTGG | ATGTTGCCGATGGTGACG | Null allele |
| NS1617 | S1617 | GTGCATTGCGTTGACTTGAA | CCGACTTATTATTTGTCCCCAG | Polymorphic |
| NS1618 | S1618 | TCGCAGCAGAAATGAAATAGAC | ACAAGGCGGAAAGATGTAGTG | Confirmed |
| NS1620 | S1620 | TGGACATCATCAGTCATTACAGC | TACATACCAGCCAAGTCATCG | Null allele |
| NS1621 | S1621 | ATTGCGGAGTAGAACTGGACC | AGTGGGCTGCCTATGGATTA | Null allele |
| NS1622 | S1622 | AACAGAACATCATCAAGCATCC | CGTCCACAAATCTAGGCAATAA | Null allele |
| NS1704 | S1704 | GAAACAACAAACCGCAACTG | AAGCAAGGGCAAAAGTGAAA | Polymorphic |
| NS1704-1 | S1704 | CTCATTGAAACACGCACGC | AGTAGTAGTCCCGCAGTAGTCG | Confirmed |
| NS1705 | S1705 | TTTATCGCTCCTGAAATCCC | GCTGTGCTCAACAACCCAA | Null allele |
| NS1706 | S1706 | GAGAACCGTTGAGTGCGAA | ATGGTCTGCTGGGAGGGAT | Polymorphic |
| NS1708 | S1708 | TCATCCAGGGTCGTCG | CTCCGTTCTGTATCGTTCTT | Null allele |
| NS1712 | S1712 | GAGAACATCTGGCAAGGCA | AGGGGAGCAATCGCACTA | Null allele |
| NS1713 | S1713 | GCGTCAAACAAGAAGCGTG | CAATACCTGAGCCTTATCGTCC | Confirmed |
| NS1714 | S1714 | TTGCTTACGAACGAGTGTCTG | TCGGGAATTTGTGGCTTT | Null allele |
| NS1715 | S1715 | AGCCTGCCTACCTTTAACTACA | AAGAACAAAACCGACATCACTG | Null allele |
| NS1716 | S1716 | TCCTATTGCCAAGCATTCAC | TCTTCAAGTCTGCCCTCATTT | Null allele |
| NS1717 | S1717 | ACCTCGTCCAAACTCTTGATAA | AACACGCAAAACACTCCCA | Null allele |
| NS1717-1 | S1717 | ATCGTAACCTGCCTCACCAA | CAATCAACGCTGTCCCAAG | Confirmed |
| NS1801 | S1801 | TGGAGACAGTTGGTTGACGA | TGAGAAGGTTGCTACATTTGG | Null allele |
| NS1803 | S1803 | AGTAAACTCCAGTCGTGCGTC | GCGTATTTGCGTTGTAGGTG | Null allele |
| NS1803-1 | S1803 | TCTGTAGGTGAAGGAATGTAGCA | CAGCCGTTCTCAAGTTTTAGTG | Confirmed |
| NS1804 | S1804 | AGGGAAGAGGTCTACCGAAG | CATTTTGCGAGTGGGCTG | Polymorphic |
| NS1806 | S1806 | ATCGTCAAGTCCCACATACCC | CGAAGAATGGCACTCATCACA | Polymorphic |
| NS1812 | S1812 | TACCATCACCCCGTCTATTTC | GTACATTACGCTTGAGTAGTTTCG | Polymorphic |
| NS1813 | S1813 | CAATGAAATGCAGCATCGTT | GACGCACTTCACTGGTATTTGT | Null allele |
| NS1814 | S1814 | CGACCTACTATTGCGTTGCC | CGTTCTCATTCATCTCATCTGCTA | Null allele |
| NS1815 | S1815 | CCTTGGCTAGGTGGTGCGAT | GGAGTGAAGTTGTGCGATTTGTTT | Polymorphic |
| NS1820 | S1820 | TGTCAAAGCCTCCGTAATAGAT | TTTCTCCACCTTATCCCACTG | Confirmed |
| NS1820-1 | S1820 | TGCTTTTAGCGATAAGACCG | AGTTCGTGTACTGTGATACCTGTG | Confirmed |
| NS1821 | S1821 | ACCGCTACCTACCTACTTTTACC | TGTTGGCTGATTGATTGTCG | Polymorphic |
| NS1822 | S1822 | TCAGTTGGCAGGTCTCGCT | CGGTTTTGACATGACGCTTAG | Polymorphic |
| NS1823 | S1823 | GGAAGAGGTTGACCGAAGAA | CCAGCAAATAGGTAGGAGAAAA | Confirmed |
| NS1824 | S1824 | ATGTTTTGTCCTGACCTCTTCTC | CTCCACTAACTCTGTTGATTTGC | Polymorphic |
| NS1824-1 | S1824 | AACAAAGTAGACTGGTAGCCCTG | CGAAATACACTCCAAAACGAAC | Confirmed |
| NS1826 | S1826 | CGTAGTGCTTGTCTCGTGCT | TGTATTTCTGTGGACCTTCTCG | Polymorphic |
| NS1827 | S1827 | GCTGATGTAGGGGTTAGGTTTC | GCTTTGTTGGGTGGACGA | Polymorphic |
| NS1828 | S1828 | TTGACGATGAGCAGGGAAC | GTAAACCGCAATGGGAATG | Polymorphic |
| NS1828-1 | S1828 | CCGTCCAACTATGCCTCTAAT | CCGATGTCGTGAACAATGTAA | Null allele |
| NS1902 | S1902 | GCATCGTCCCATAAAG | GTCGCACTGGAAATCTA | Polymorphic |
| NS1903 | S1903 | CCAACTCCTCATTCCTGTCC | CCCTTAGCCTACAAGCGAAT | Null allele |
| NS1904 | S1904 | TATCCACAGCCCTAACAACG | ACCACTAAGATTCGGTAAGCAG | Polymorphic |
| NS1904-1 | S1904 | AGCGTGTAACGGTGTAAGTGAG | GGTTTCGGGCAGTTTTCG | Null allele |
| NS1904-2 | S1904 | TTCGTAGTGCCTCAATCCATAA | TGGCTTGCTTCGTTTTCC | Confirmed |
| NS1907 | S1907 | GTGCTAATGGTATTTGAGAACGC | CAAGAGTGACACGATTGAGAAGAA | Null allele |
| NS1910 | S1910 | GGTTTCCTGATACTGGTGTTCTT | GGACGGATTCCTTTTGTTTG | Polymorphic |
| NS1910-1 | S1910 | CAGCCGTTTTCCAGTTTTAGC | GGACATCAACAAGCACTTATCACA | Confirmed |
| NS1911 | S1911 | TAGTGGTCCCGCAGTAGTCG | TTCGGAAAAGGGGTGTTAGT | Polymorphic |
| NS1912 | S1912 | TCGCTCTTTTACCTTTACCGT | AGCCGTTTCCGAGATTCAG | Null allele |
| NS1912-1 | S1912 | ACACTAAAACAACTGAACGCCA | TAACTGTCCCTCCCCTCCAC | Null allele |
| NS1913 | S1913 | TCTCAGTAGGTAGAGTCTTCCGA | GTGCTTGTGCGTATGCGT | Null allele |
| NS1914 | S1914 | TGATGTGGAGGATGTTATGGA | GCGATAAGACCGCCTATTGTA | Polymorphic |
| NS1917 | S1917 | CTTACTTACCTTACGGGCTTGG | TTGATACGATACAGTTGGCAGAG | Confirmed |
| NS1918 | S1918 | ACAGAAAGGGACAAAATACGG | TAGCGAGACTAACGAGCAGC | Confirmed |
| NS1919 | S1919 | GGATTTACTAACAACTGCCCG | TTACCAGAATACCATCCCTACCT | Polymorphic |
| NS1920 | S1920 | GGGAAGAGGAGTTTCGGATT | ATGTTTCGTCGCAGGGG | Polymorphic |
| NS2001 | S2001 | TAGGGACTACTGTTTCGGTGG | GAGCGATAAAGGGCTGTGA | Polymorphic |
| NS2004 | S2004 | TCACCATCGTCATCAACACC | GCAAACCCTATCCCTCCTG | Null allele |
| NS2007 | S2007 | GCTTGTTCATTCGTCTATCCA | GGTGTTCCCATCCATTACTAAA | Polymorphic |
| NS2007-1 | S2007 | ATGGGGTTTGGATTTCTGG | TGGTAGAGCACGACATCACAG | Confirmed |
| NS2008 | S2008 | GCTGTTAGCAAATCCCACCC | CCTTCTACCTTGTCTCCACCTTC | Confirmed |
| NS2010 | S2010 | TTGTGGATAGTCCCTCCTTTG | CAGACTTCGTAGTGCCTCAATC | Polymorphic |
| NS2014 | S2014 | AACGAGGCTTGCGTAGAGTG | GGATAGAAGGGCAGGATAATGA | Polymorphic |
| NS2015 | S2015 | GCTTACGAACGAACGGTGA | CTGAATGATACGGTGGTGCTA | Polymorphic |
| NS2015-1 | S2015 | GCCGATGTCAAATGAAAAGA | CGAAACCTACGAATCAGAAGAA | Null allele |
| NS2019 | S2019 | GTCGGCAGATGACCACAGT | AGCCCCTATCGTTACAAGAAG | Polymorphic |
| NS2020 | S2020 | CCTGCGAGACCTTTGATTTT | CACTTACCACTACACCAGACGG | Polymorphic |
| NS2024 | S2024 | AACTTTTACACTATCGGCAACG | CAGGTGACTGGGAGAATGACA | Polymorphic |
| NS2025 | S2025 | AGCGGTTAGTTGCCCTCA | CGGCTACTTCCATTATTCATTC | Confirmed |
| NS2026 | S2026 | TAATACACCAGGGGACAAAACA | TTGGATTGACCTGGGAAGTG | Confirmed |
| NS2026-1 | S2026 | CTTCTTTTACTCCACCTTATCCC | CATTTGTTACCGTTTGCCG | Confirmed |
| NS2102 | S2102 | GGTCCTATTGTTACAGATTTCGC | AGGGCGAGACATCAGTTACA | Polymorphic |
| NS2104 | S2104 | CGACAATCCTATTTAGAACTCCTG | GGTGTTTTCCTCCATTCCTTAT | Null allele |
| NS2105 | S2105 | GCGGGTCTAAGTCTGGTCA | TCTGTGGAAGGCATTGGG | Polymorphic |
| NS2106 | S2106 | GTTCGTTCCCATACTCCTCC | ATTAGCCAAATCGGTCCAG | Polymorphic |
| NS2107/08 | NS2107/08 | AAATACTGTCCTCCACTATCCCA | CAGCAAACCCAGACATAAACAA | Polymorphic |
| NS2107/08-1 | NS2107/08 | TGTGATAGGGTAATGATTGGGG | CAGCCGTTCTCGGGTTTT | Null allele |
| NS2109 | S2109 | TGCGTATCATCAGACTTCGG | GTCTGTATCTACGGCAGTTAGTTTT | Null allele |
| NS2112 | S2112 | GCTGAGTGAGTAATCCGAAACA | AATGAAAGTCTGATGCCGAA | Null allele |
| NS2113 | S2113 | CTTATGGGACGATGGGGA | TCGGGATGATTTATGATGCT | Polymorphic |
| NS2116 | S2116 | AATACGAAGCACCTTTCCTGT | GCCACGATTGTTGATTGTTG | Polymorphic |
| NS2117 | S2117 | CAAAACCATCCGAACCACA | CACGATTACAGGACTCCATACAT | Polymorphic |
| NS2118 | S2118 | TCTTGTAAAACTGCTGTCTCATCC | TATTCGGTCGTGTTGTGGTAG | Polymorphic |
| NS2118-1 | S2118 | GGAGAAGATAGGGATTTTGCTG | AAGAGGTGGTGTCGTGGGA | Confirmed |
| NS2119 | S2119 | AATCCATTGGGAACTGAAGC | CCTGTCGAAGGTCGTCTGA | Polymorphic |
| NS2119-1 | S2119 | TGACCACAGCAATAAGAAAAGC | CGAGCCTAACGAACAGCAA | Confirmed |
| NS2201 | S2201 | TTGAAGGTGAAGGAGTTTTGTG | GGTTTTAGTTAGCGTAGGGTGTT | Null allele |
| NS2203 | S2203 | TAACAACCCTCTTCCCTACGG | CCTCTTCAATGTGATGCTTCCT | Polymorphic |
| NS2207 | S2207 | ATGGTGTATCTTATGTGGAAGTGTC | TGGCTGGGCATTATTTATTG | Polymorphic |
| NS2208 | S2208 | GTTGGCTTGGTGTCTTTAGTTG | CCCTATCGCCTTCTATGTCA | Null allele |
| NS2209 | S2209 | TAGGAGATGTAAGGAAACGGTAG | CTGGGAACAAATCACGCA | Polymorphic |
| NS2212 | S2212 | GCAGGGTCGGGAATGTAGT | AGAATGCTTATTGGCAGGAAC | Polymorphic |
| NS2212-1 | S2212 | AACAGTCTACCTTAGGGGAGTGC | ACGACACGGCGACATTGAC | Confirmed |
| NS2214 | S2214 | GTCCCTACCTACAAGATGGCA | CAAAAGGATGGTCAACGCA | Polymorphic |
| NS2214-1 | S2214 | AGCGTCTCAAAGTAAAGGGG | CGTTCGGTGCAATCGTGT | Polymorphic |
| NS2215 | S2215 | TTTTGATGAGACCAGAAACAGG | GGGATTCCAACACCGACA | Polymorphic |
| NS2219 | S2219 | AACCGATAACCGTTGGGG | TTTGAAGGGCGAGGCAG | Polymorphic |
| NS2220 | S2220 | GTCAGTTGCGTTTTGTTATTCC | GCCTCACTTCGTCTCCTCAT | Null allele |
| NS2221 | S2221 | GAAATAGCCGAATAGTAGCCAG | CCCGCCAAGGGAAGATA | Null allele |
| NS2222 | S2222 | TGGGTGCCTCATAAGCGT | AATCGGTCCAGTAGTTTCGG | Polymorphic |
| NS2222-1 | S2222 | CTCGCTGGTTTGTGCTTTC | TGGGGTGGAGACTGGGATA | Confirmed |
| NS2223 | S2223 | TTCTCCGCTACTGGTATGGC | TGTTTGTGGAACGGGTCG | Confirmed |
| NS2224 | S2224 | TAGGCAGACGAGCATACGG | CGCACGGCAGATCATTG | Polymorphic |
| NS2225 | S2225 | AATGAATTGGCAAGCGAGTA | AATGAAACGGTTGTCTGGAAG | Confirmed |
| NS2225-1 | S2225 | CGGTTCGGTTGAGTTGAGA | CGTGGGAATGAGGCGTT | Null allele |
| NS2305 | S2305 | GGTTGGAAGGATGCGACA | GCCCGAAGAACTTGAGGAT | Polymorphic |
| NS2305-1 | S2305 | GGTCGTCTCGTCTCCTCATC | TGCGGCGTGTAGTATTGTG | Confirmed |
| NS2307 | S2307 | CCGAAAGTGACCCTTAGCA | CCAACTATTCAACTGGACAACC | Polymorphic |
| NS2308 | S2308 | CATTACCAACGAGTCAAAACG | GAAATGGGCAGGTTGGTG | Polymorphic |
| NS2308-1 | S2308 | ATTAGGTGATGATAGACGCAGG | ATTTTGTATGGGCATTCGC | Polymorphic |
| NS2310 | S2310 | AGCGACCTGTCAGAAAGCG | CAATAGAGCCGTTACAACCAATC | Null allele |
| NS2311 | S2311 | CGCAAGCCGTAGAAAATCG | ACAATGGACGCTGAGACCG | Null allele |
| NS2311-1 | S2311 | GTCAAATCCGCATTATTACGC | CAGTCGCAACCGCTACCTA | Confirmed |
| NS2312 | S2312 | TCCTCCAGTCCAGATTTGAA | CGAGACATTAGTCAAGGGATTAC | Null allele |
| NS2314 | S2314 | ATTCCCATCCCGCAACTG | GGTCTTATCTACATTACGCCACAAA | Null allele |
| NS2314-1 | S2314 | ACAGTGTAGCCTTAGCGAAATC | CCCTTTATCAGTGTAAGTTATCCG | Null allele |
| NS2315 | S2315 | CTATCACTTTGCCACGGACA | GGGCTGACGAATACCTAGTTTAA | Null allele |
| NS2316 | S2316 | CAACGAACTCGCCGTATCT | TTCCAAGTCGCCATTTAGC | Polymorphic |
| NS2316-1 | S2316 | AATAACTTGCTCCTAACTTAACGGC | TCGGGACACGCATCCTACA | Polymorphic |
| NS2316-2 | S2316 | CATTATTGGTTCGTTCTTATCGTCC | AGAGCGGGTGCTTCTCCTGT | Confirmed |
| NS2318 | S2318 | GTAAATGATTCTTGTCCACCTGATG | ATTAGCCGACCCAACCCTG | Polymorphic |
| NS2319 | S2319 | TTGTTTACGTTTTGTCTGGGTG | TTCATACTCCTCCGTCCGC | Polymorphic |
| NS2320 | S2320 | GTTCAAGATAGGAGGATGGGTT | GGACAGCGGTAGAGCAAAA | Null allele |
| NS2321 | S2321 | ACCCACTCACGAAACTCAATG | TCAAACAGCCCTAACACCAA | Polymorphic |
| NS2322 | S2322 | GTGGACACTAAAGTTCGTCATTG | GTGGACCCCATACAACATCTT | Null allele |
| NS2323 | S2323 | GTTCACGATTATTGAGGCATTG | TGAGGAAACGATTTTACAGGG | Confirmed |
| NS2325/30 | NS2325/30 | ACGAAAATAACCTGACACCCC | CCCCTCTGTAAAACTGAATGAA | Polymorphic |
| NS2325/30-1 | NS2325/30 | TCCGATAACCACTTAGCAACA | GAGCGAGGAACAAGCGATA | Confirmed |
| NS2329 | S2329 | AACAACACGGTCGGTATTAGG | CGGGCTTTTGCTTTCCA | Polymorphic |
| NS2329-1 | S2329 | TCAATGAACGACAGGTGGC | TGGCTGGGTAATCTAGGCG | Confirmed |
| NS2331 | S2331 | TCAACACGAACAGCGGC | CTACGGGTTTTACCAAGCG | Null allele |
| NS2332 | S2332 | TACCGAATGCTCCCTGACG | TGCGTTCATTGGCGTGG | Confirmed |
| NS2333 | S2333 | CCCGCATCCATGTTCCA | CCCTCTTGCTTGTGATTTGTTA | Polymorphic |
| NS2333-1 | S2333 | TCGTTTGGTAGGGTATGTATGAG | CGCTGTGACAGGTCCTTCTT | Null allele |
| NS2334 | S2334 | CGTGGGCTATGAAATGGAA | CAATGTTGCTGACGAGGATG | Confirmed |
| NS2335 | S2335 | TCTGGAAACGCCAACTCG | GCCTTTATCTCGGACGCA | Polymorphic |
| NS2335-1 | S2335 | GGACACCATTAACCACTTGACA | CCGCCACCTACTTGAGACA | Polymorphic |
| NS2406 | S2406 | AAAGTCCAGACAGACGAGCAT | TTGTGAATCTAAACCATCCAGG | Polymorphic |
| NS2406-1 | S2406 | TTTTCAACCCTTACCTACGACC | AGCGAAGTGGAGGAGTAAAGTC | Confirmed |
| NS2408 | S2408 | CCATCTACAAAAGAAGCAGTCA | CAATTACCAGCCAACAAAGC | Polymorphic |
| NS2409 | S2409 | GTAATGGCGTCAGTCAGTCC | GCACAACGGCATAGTGTAAGA | Confirmed |
| NS2412 | S2412 | CGAAGTAGCAAAACGAGGAA | GTGTAAAATCGGAAAGGCATCT | Polymorphic |
| NS2414 | S2414 | TGTAGCCAGTCCGATTGTAGC | CATTATTGTCCACCTTATGGTTGTC | Polymorphic |
| NS2416 | S2416 | TGAAAATGATTCGCACGGT | GGAAGTCGCCTCTAATAATACATAC | Null allele |
| NS2417 | S2417 | AAGAGTCGGGGAGTAGTCAGTG | TCGGCGATAGATGGCGTT | Polymorphic |
| NS2417-1 | S2417 | TAAAAGCAAAACCTTAACTGTCCCC | TTACACCCCTCACTCCGCCTAC | Confirmed |
| NS2418 | S2418 | AAAATGAAGGGTAGACTCCGTAAC | CAATGACAGGATAAACCGACAG | Confirmed |
| NS2420 | S2420 | GTTCTGAGCAACCTTATGGACG | TGTCCTTTTCTACCTTCACGA | Null allele |
| NS2422 | S2422 | TCAAGAAAACTTCCTTCCCAC | TTCCCGCCTCAGCAATAG | Null allele |
| NS2424/25 | NS2424/25 | CCATCTTGGTATCCTCGTCG | GCCGTCATCTCAACACTCACT | Polymorphic |
| NS2424/25-1 | NS2424/25 | ACGAATGCGAGACATAGTAACG | CTCCCATACAAGAAGGTGCTC | Null allele |
| NS2426 | S2426 | ACCTAAACGACGACCTAAACCT | TGTCCTACAGAGCAATCAAAGAGT | Confirmed |
| NS2426-1 | S2426 | GATGGAGACAGTTGGTTGACG | GGTGTAAGAGTGCAGGGGAG | Confirmed |
| NS2426-2 | S2426 | GGTCCTATCTTGTCCACATTCC | CTATCTTCTTCTTCTTTTCCTCCAC | Confirmed |
| NS2430 | S2430 | CATCTCGCTCTATTCGGTTATC | CAGGGACAATGTCGGCTT | Null allele |
| NS2431 | S2431 | CCTGGTGGTTGTCTGGAAG | GCATTCTTTAGGGGCATTGA | Null allele |
| NS2432 | S2432 | AGCGAGACTAACGAACAGCA | TCCACCCAACTAACCAATAAAC | Null allele |
| NS2434 | S2434 | GCGTCATCAAATGCGTAAAA | TGAAAATCGTTGGAGCCGT | Polymorphic |
| NS2436 | S2436 | CCCCACCGCTAATAAATCC | CCGCTGCCTACCGAAAT | Null allele |
| NS2437 | S2437 | CTAAACCACGCCCGTCTCA | CAGCCGTCATCTCAACACTCA | Confirmed |
| NS2438 | S2438 | AAAAGTGAGTGTATGTGGCAGAG | CGTCAACGATGAACCGAGA | Polymorphic |
| NS2440 | S2440 | CTGTCCATCCCGAACTCTAATT | ACGGAAGATCATCTCCAACG | Confirmed |
| NS2441 | S2441 | TCTCCTATTTACCTTCACAGCG | CAACTCCCTACAACTACCCTCA | Polymorphic |
| NS2442 | S2442 | TTTGTAGTTTTAGTGGGGTGCT | GGTGCGGAATGGGAAGA | Polymorphic |
| NS2442-1 | S2442 | TCTGGACTAAATCTTCGTCTGTG | TCGTTTGACCCTTGCCTC | Confirmed |
| NS2443 | S2443 | AGTAGTCAGTGTCTGGTGCCC | CTTGTCCCTCTTGTCTCGGTA | Null allele |
| NS2445 | S2445 | TGTAAGCGAGTGTAAGCGTGT | TCCCGTCTTGGATTAGGTGT | Confirmed |
| NS2446 | S2446 | CGTGGAACGGTGAAGATGA | TGATGACTTGGGAGTTTAGGAA | Polymorphic |
| NS2446-1 | S2446 | AGCGAGACTAACGAAGAGCAA | CGGGGCAACACCAACAT | Polymorphic |
| NS2501 | S2501 | GGCGGACAGGAGGTAGT | TTGGAGGTGGTTAGCG | Polymorphic |
| NS2502 | S2502 | ATTAGTCAACAGACAAAGACCAGTG | GATGTAAATAGGGACGGCAAC | Polymorphic |
| NS2505 | S2505 | GGATTCTGTCTAACCCTTCA | ACCTGCCGCCTATCTATT | Null allele |
| NS2506/07 | NS2506/07 | ATGGGTATCACCGTCCTTCC | CGGGCTTAACTTGACTTGCTA | Polymorphic |
| NS2510 | S2510 | GTGGATGAAAGTGACGAAACA | TCAGGGGAGTGATGAAAGAA | Null allele |
| NS2511 | S2511 | CCAACGGATGAGTCAGAGTG | CTTGTTTTATTATCGCCCCA | Polymorphic |
| NS2515 | S2515 | GCTTATGTCGTTTAGGCTTCG | TTTCACCCGTGCCCAATG | Null allele |
| NS2517 | S2517 | TGCTCGTCAGAATACAAGGG | CACCGCTTTCAGATTCACAA | Polymorphic |
| NS2519 | S2519 | GTAAAGATGCGTCTACCAGGG | AAGCGAAGGCAAAATAAGC | Polymorphic |
| NS2520 | S2520 | ATAGAGTAGGGGAATCCACGA | GAATCCTTTGTCACCTCCTTG | Polymorphic |
| NS2521/22 | NS2521/22 | CACGCTCTTATCTTACTCGC | CACCACTACTGACAAGGGAC | Null allele |
| NS2530 | S2530 | TACTAATGGTTGCCCGCC | TGTCTTGTGCTACTGCCCG | Confirmed |
| NS2532 | S2532 | TTCGCTTTGTCGTGTCTATGA | AATCGTTTGAAGAGTTGGAGTG | Polymorphic |
| NS2532-1 | S2532 | CTGCCTTTATTTGGTCGGTC | CATCTTTGTTACGTTCTGTGCC | Polymorphic |
| NS2534 | S2534 | CCAAATACAGGAATAGCGAATG | AAAGGGTCCCCGAGAAAG | Confirmed |
| NS2534-1 | S2534 | GCCATAGTGAACTCCATATCTCC | TTTCCTCTACCAGCCCTCG | Confirmed |
| NS2601 | S2601 | CGCCCCTACAAACAATAACTA | CTCCGCATCGTCCCATAA | Polymorphic |
| NS2604 | S2604 | CAGTCTCATTTCCACTCGCTA | TCGGTGCTTTAAGGCTTCT | Null allele |
| NS2606 | S2606 | TCTATGGGCTCTTGTCACTATTTG | TGGGTCTGATTGGGTTTCG | Polymorphic |
| NS2607 | S2607 | TGTAGGATTGGTAAGAACGGAA | CTGCTAAATGCTCACGAAAGA | Null allele |
| NS2608 | S2608 | CCACAATGGTCGCAACAC | CATACGAGTAAGTCTCCTCACAAAT | Null allele |
| NS2609 | S2609 | GTGAGATTTCAAAAGCAAGGC | GAACTACGAAGTCTGCTGGGT | Polymorphic |
| NS2610 | S2610 | TACCACTGGGTCGGAAAC | TCAAGGGACACGAAGATG | Polymorphic |
| NS2612 | S2612 | TTATCACCATTGCTCTATCCG | TCCGTCCGTCTCAGTCAGT | Null allele |
| NS2614 | S2614 | ACTCATCTACCCACTAATGTAAAGC | AAGAACCAAAGGGAAACGG | Null allele |
| NS2615 | S2615 | TCTGCCTCCGCATAATAACT | TTCCCCTTTCTCAGTTCAGTC | Null allele |
| NS2616 | S2616 | GCGATGAGAAAACAGGAGC | AATACAAGCCGAGCACCAA | Polymorphic |
| NS2617 | S2617 | TCTAAGTTCAATCACGACCCC | GCGAGACTAACGAACAGCAA | Polymorphic |
| NS2625 | S2625 | ATTGCTCGCTGTTTCTTGG | TCCCTTCCTCTAAGTCTCGG | Polymorphic |
| NS2626 | S2626 | CGCTGGTGTAATGTTTGTGAA | TTGGCTTAGTCCTCCGTGA | Confirmed |
| NS2626-1 | S2626 | GGTTACCGTTGCTTGTGGA | CCGATTTCGTGGACTGTTG | Confirmed |
| NS2627 | S2627 | GTGAAGCAGTAATGCCTTTTGG | GCTCCGAGTTTCCGTCATAGTA | Polymorphic |
| NS2627-1 | S2627 | TCTGTCTCATTCTTTGCTGGC | CGAACTTCGGAATTGGTTTTAC | Confirmed |
| NS2629 | S2629 | GGTCGGCGTCAATCCAA | TAGGCAACCCAAAACTCACA | Confirmed |
| NS2630 | S2630 | GCCGTTTAGGAGGATTTCAG | GTTCAGCCACAGTGCGTTT | Polymorphic |
| NS2701 | S2701 | CCGCAGTAGGTCGGTATTC | CGGAGACGGAGGAGTATGA | Null allele |
| NS2704 | S2704 | TCACAGCAGACCAACAGGAA | TTATCGAAAAGCAATCACGC | Polymorphic |
| NS2705 | S2705 | TAGCACTCTAATCAGAAGACCCTC | AAACACGAAACCGAACCG | Null allele |
| NS2707 | S2707 | ATTCCGCATCAGGTTTAT | GCAGTCACCGTTCCAGTA | Null allele |
| NS2708 | S2708 | GTTTTGGGACAACTGTATTAGCC | GGAGCCGTTTCCGAGATT | Polymorphic |
| NS2712 | S2712 | CGACAGGAAACCCAAATAGG | GATAAATCAGTAAGTCAGACCAAGG | Null allele |
| NS2714 | S2714 | CTGCGAGGGAAGGGATACT | TGTGGCTGCGAACAAGAAG | Null allele |
| NS2716 | S2716 | CGACAGGACAAGTCTACGATTAT | AACCGTTTCAGAGGAGTATGG | Null allele |
| NS2801 | S2801 | CGCTCCCCTAAATCTACCAT | ACCAACTTGCCTGTTTCTCC | Polymorphic |
| NS2801-1 | S2801 | GGCTCGTGCCATAAAATCG | TCCTCCTCCTCCTTCAGTCG | Null allele |
| NS2802 | S2802 | CCTTCCTATTCTCCGTGTA | TTGGTCGGTCTGATGC | Polymorphic |
| NS2805 | S2805 | TGTTTATCTTTCCGCAGGTT | TGTTCCGTATGTGCTCAATGT | Null allele |
| NS2806 | S2806 | ATACCCGTCCACTTTCACTCT | TTTAGCCGTTCTGATTCGTT | Null allele |
| NS2808 | S2808 | GATGCGGATTCGTTTGCT | TGTCTATGGTGCTTGAGGGG | Polymorphic |
| NS2808-1 | S2808 | CTCTGCGTGGTATAGGGTTGA | GAGTTTCTGTAAGCACATCGGA | Confirmed |
| NS2809 | S2809 | AGCGGTTTACCTGGAAGCA | AAAAGAAAAGAGGCACGACG | Polymorphic |
| NS2813 | S2813 | CAATGTGGTGGTCGGTGA | GATTTCGCCTACAGACTACACTT | Null allele |
| NS2814 | S2814 | GACGGCGATAGGCTGAAA | AGTAGGATAGAGCAAGATGGTGG | Null allele |
| NS2814-1 | S2814 | AGGGCGAGGTAGAGGAGAA | TGAGCGACAGGAGGTTTTAGT | Null allele |
| NS2815 | S2815 | AGTCAAAACTCAAAATCCAGCC | GCCTTCAAGTGTTCTACTCATTCTAC | Null allele |
| NS2816 | S2816 | CGTCTCACCAGTATCCACCG | GTTCGTCCAAACATCTAAGCA | Confirmed |
| NS2816-1 | S2816 | AGCAGCAGTAAGCCTTGGAC | GTGAGGATGTTGTTTGTTTGTAGAG | Confirmed |
| NS2816-2 | S2816 | ATGCGGGTGTAGTATTGAAGAA | CCTATGTAACTGGAAGGAAGCG | Confirmed |
| NS2816-3 | S2816 | TCCGAGTTTGTAAGTCTGTTTCTG | GTGCTTTAGTATGCCAATAGTGATG | Confirmed |
| NSXX01 | SXX01 | CGTGAAGAGCGTATGCCCA | CCCTACCACCACCGTGAAAT | Null allele |
| NSXX02 | SXX02 | TTGAGTGCGGGAAGTTGG | ACCGTTTACTGGATAGGGCA | Polymorphic |
| NSXX04 | SXX04 | CAGATATGCCGTGAGGTCG | CAATCGTGAGGTATGAGCAGTAA | Polymorphic |
| NS0308^1^ | - | GGTCGGTCTTGGGTTAT | TCGGTCGTGTTCCTTG | Confirmed |
| NS0308-1^1^ | - | GAACGCAAGATACTGGTGGC | CGTACAAACTCATTTCAGGCAA | Polymorphic |
| NS1027^1^ | - | TTGTTCGTCGTTTGTTCGTT | CTTTTAGGCACCACAGGCA | Confirmed |
| NS2204^1^ | - | CGTTTACCATCTACCTTTCCTG | TCCTTTCGCACTGTCTTTTG | Polymorphic |
| NS2204-3^1^ | - | ATGATGACACTCGTATTGTTGGC | CGGGTGATATTGCTCGGTAGA | Polymorphic |
| NS2204-1^1^ | - | AAGATTAGCAGCGACATTCACA | GGTCCAGCCATCCTCAGTTT | Confirmed |
| NS2204-2^1^ | - | GCGAAAGAGCAACACCGT | AAACGCCAACAATACGAGTG | Confirmed |
| NS2122^1^ | - | CATCGTGCGAAGACAAATAAG | CTTTAGACAGGTCATCCTTCCAT | Polymorphic |
| NS2328^1^ | - | CCAAGTTTCAAGACTCCACGC | TTGTTCTAAGTAGGACACGGCT | Confirmed |

1 These nine neighboring markers were not included in the integrated map, but they were involved in the correlation analysis between SSR marker type and development efficiency.
