# Supplementary material for: An integrated genetic linkage map for silkworms with three parental combinations and its application to the mapping of single genes and QTL
Source: BMC Genomics. 2009 Aug 21;10:389. doi: 10.1186/1471-2164-10-389 (PMC2741490; doi:10.1186/1471-2164-10-389)
Supplement: Additional file 16 — Comparison of the efficiency of different mapping populations and marker types. This table contains the origin of three populations and the counting information on markers used in each population. [file 1471-2164-10-389-S16.doc]

## Comparison of the efficiency of different mapping populations and marker types

|  |  |  |  | Effective Data Set | | Mapped Sites | |
| --- | --- | --- | --- | --- | --- | --- | --- |
| Cross | Origin | Marker type | Total employed | Total | Previous^1^ | Total | Previous^1^ |
| DC^1^ | China/China | SSR | 2,500 | 555 | - | 518 | - |
| JL | China/China | SSR | 890 | 119 | 80 | 94 | 51 |
| NF | France/India | SSR | <2,500 | 271 | 109 | 251 | 100 |
| NF | France/India | Gene-based | 225 | 16 | - | 12 | - |

1 “Previous” indicates the set of markers included in our previous work [7].
